# Traditionally Used Edible Flowers as a Source of Neuroactive, Antioxidant, and Anti-Inflammatory Extracts and Bioactive Compounds: A Narrative Review

**DOI:** 10.3390/molecules30030677

**Published:** 2025-02-04

**Authors:** Maciej Książkiewicz, Michalina Karczewska, Filip Nawrot, Katarzyna Korybalska, Elżbieta Studzińska-Sroka

**Affiliations:** 1 Section “Pharmacognosy”, The Student Scientific Society of Poznan University of Medical Sciences, Rokietnicka 3 Str., 60-806 Poznań, Poland; 2 Department of Pathophysiology, Poznan University of Medical Science, Rokietnicka 8 Str., 60-806 Poznań, Poland; 3 Department of Pharmacognosy and Biomaterials, Poznan University of Medical Sciences, Rokietnicka 3 Str., 60-806 Poznań, Poland; elastudzinska@ump.edu.pl

**Keywords:** therapeutic potential, phytochemicals, traditional medicine, edible flowers

## Abstract

Edible flowers are becoming a popular addition to diets. As science has progressed, it has been proven that in addition to their aesthetic value, they possess pharmacological effects and health-promoting properties. Several edible flowers are used in medicine, and the available literature data indicate their broad biological activity. This review focuses on pharmacological knowledge about the neuroactive, antioxidant, and anti-inflammatory potential of 15 traditionally used edible flowers. It also describes their traditionally uses and summarizes research findings on their chemical composition.

## 1. Introduction

For centuries, plants have served as a vital source of drugs for therapeutic applications [1]. In this context, not only entire plants but also specific parts such as leaves, roots, and flowers have been utilized. Flowers have played a significant role in traditional medicine, being used in remedies for ailments such as inflammation and digestive issues and relieving excessive agitation, among others. For example, calendula and chamomile flowers were used externally to treat skin inflammation [2,3], while elder and mullein flowers helped treat common cold and coughs of various origins [4,5]. In addition, lavender flowers were used for calming purposes [6].

Apart their medicinal applications, flowers have also been incorporated into culinary practices across various cultures [7]. The use of edible flowers has been also noted in Asian, European, Indian and Middle Eastern cuisine and was directly connected to local festivities and culture [8]. Calendula flowers were used in medieval France as the addition to salads, rose petals were chosen by the ancient Romans to sweeten their dishes, *Viola odorata* flowers used to be an important food colorant and in Central Europe instead of honey locals used a mixture of sugar and elderberry or dandelion flowers [9].

Scientific studies emphasize that edible flowers, traditionally used as an aesthetic addition to food or as a vegetables, are not only a source of nutrients but also have a valuable phytochemical profile [10]. The main chemical component of flowers is water, which constitutes 70–95% of their total content [11]. Moreover, the dry matter of edible flowers consists of carbohydrates, proteins, lipids, vitamins, minerals, and low-molecular-weight phytochemicals [11,12]. Flower-derived chemical material can be divided into three main parts: pollen, nectar, and petals. Pollen is a source of carbohydrates, proteins, lipids, carotenoids, and flavonoids. Nectar contains sugars, proteins, lipids, organic acids, and secondary metabolites such as phenolic compounds, alkaloids, and terpenoids. Petals, in turn, are rich in vitamins, minerals, and various polyphenolic compounds known for their antioxidant properties [9].

**Figure 1:**
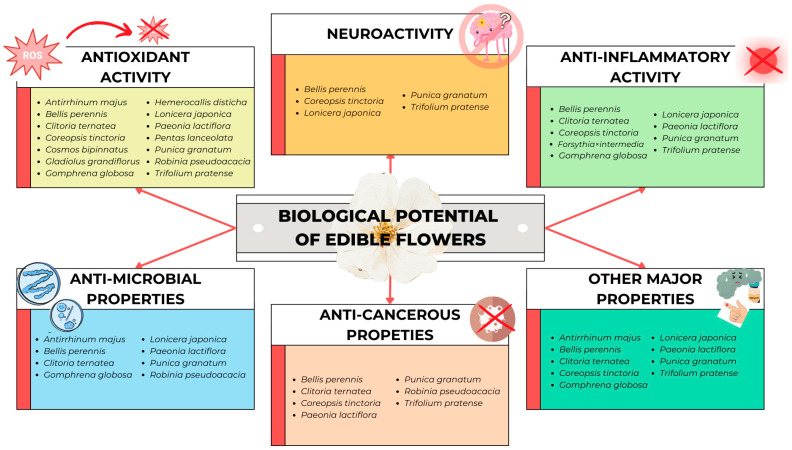
Biological potential of edible flowers.

Studies have shown that edible flowers have significant pharmacological properties [13] (Figure 1). This bioactivity is correlated with their rich chemical composition, which contributes to the wide range of functional properties of edible flowers [14]. Edible flowers can therefore be seen as a source of compounds important for disease prevention. They are an excellent source of antioxidants, which are considered effective free radical scavengers. These antioxidants play a fundamental role in protecting humans against many degenerative and stress-related disorders, including neurodegeneration and inflammation [15].

Consequently, the popularity of edible flowers continues to rise, as they are increasingly perceived as a novel way to enhance dishes with vibrant colors, non-toxic nutritional benefits, and the potential for indirect disease prevention [15].

The aim of this paper is to demonstrate that edible flowers are not only decorative elements in culinary but are also a source of bioactive compounds that contribute to their significant health-promoting potential. This article focuses on how the traditional use of 15 selected edible flowers corresponds with current pharmacological knowledge regarding their neuroactive, antioxidative, and anti-inflammatory potential.

## 2. Results and Discussion

### 2.1. Botanical Characteristic

In this research, we focused on 15 plants that are proven to have great neuroprotective, antioxidative and anti-inflammatory potential. The list of the reviewed plants and their short characteristics are presented in Table 1.

### 2.2. Traditional Medicinal and Dietetcic Uses

For thousands of years, flowers have been valued for their therapeutic properties [45]. In traditional medicine, edible flowers were believed to exhibit a wide range of medicinal activities, including anti-anxiety (e.g., *H. disticha* [46]), anti-cancer (e.g., *T. pretense* [47]), anti-diabetic (e.g., *G. globosa* [48]), anti-inflammatory (e.g., *A. majus* [49]), antioxidant (e.g., *G. globosa* [48]), diuretic (e.g., *B. perennis* [50]), anthelmintic (e.g., *P. granatum* [51]), immunomodulatory (e.g., *T. pretense* [52]) and antimicrobial effects (e.g., *G. globosa* [53]). According to research conducted by Lu, Li, and Yin (2016), the use of edible flowers spans 97 families, 100 genera and 180 species [54]. An analysis of the traditional uses of 15 selected species of edible flowers reveals their application in treating a variety of ailments.

The most commonly documented applications in the scientific literature pertain to disorders of the digestive system, particularly liver function and intestinal issues [49,55,56,57,58,59]. Additionally, edible flowers are frequently cited for their diuretic properties, supporting the treatment of urinary system ailments and hypertension [9,51,60]. They have also been utilized in the management of respiratory dysfunctions, including symptoms of asthma, bronchitis, and cough [47,51,61]. Other reported benefits include their effects on the nervous system, such as relaxing nerves and muscles, providing a sedative effect, and alleviating symptoms of depression [50,57,62]. Furthermore, edible flowers have been recognized for their anti-inflammatory and antimicrobial properties [14]. A detailed overview of the traditional uses of the selected edible flowers is provided in Table 2.

In addition to their use in folk medicine, the flowers described in this paper have been traditionally used in the preparation of various foods or beverages. The flowers of *C. ternatea* were used as a food dye and vegetable in South East Asia (India and Philippines) [59,63], similarly to *Gladiolus grandiflorus* flowers [64]. Indian tribes, on the other hand, used the flowers of *C. tinctoria* to prepare an infusion in the form of a tea. In East Asia and China, the flowers of *L. japonica* and *P. lactiflora* were used to prepare beverages [65,66]. *P. lactiflora* was used as a base for making wine. It was also one of the additives to porridge [67]. In turn, in East Asian countries (e.g., Taiwan) the flowers of *H. disticha*, both fresh and dried, were used as an ingredient in various dishes, including vegetarian dishes [11,68]. In Europe, the flowers of *R. pseudoacacia* were used in both fresh and cooked dishes. The flowers of *R. pseudoacacia* were added to pancakes and also prepared in a sweet coating. In Italy they were used to flavor grappa, and in Spain they were chewed or sucked as a substitute for sweets [69].

**Table 2:** Traditional medicinal uses of chosen flowers.

Plant Name	Place of Use	Traditional Medicinal Use	References
*Antirrhinum majus*	Iraq	detergent, astringent, diuretic, and for treating liver ailments (decoction from a whole plant), resolvent, stimulant, and anti-inflammatory agent	[49]
	Other countries	gum scurvy, ulcers, hemorrhoids, diuretic, treating scurvy, astringent, antiphlogistic, stimulant, tumours and ulcers, inflammation, treating watery eyes	[55,56,70]
*Bellis perennis*	Turkey	antispasmodic, sedative, carminative, diarrhea, diuretic, purgative	[50]
	Brazil	stomach problems and clamant	[57]
	Austria	respiratory tract and gastrointestinal diseases	[58]
	Other countries	expectorant, diuretic, anti-inflammatory, vulnerary, wounds, rheumatism, eczema, eye diseases, inflammation, tonsillitis	[71]
*Clitoria ternatea*	Indonesia	eye disease, fever, cleaning and clearing baby’s eyes	[72]
	South Asia	boosting memory and improving intellect, mental illnesses, rejuvenation in neurological disorders, eye infections, headache	[62]
	India	snakebite and scorpion sting	[59]
	Cuba	emmenagogue, vaginal douche, liver and intestinal problems	
*Coreopsis tinctoria*	Native American tribes	panaceum (Navajo), reproductive aid drugs (Zuni), antidiarrheal (Cherokee)	[73]
	China	clearing heat and detoxification, promoting blood circulation and removing blood stasis and spleen, hyper-tension, palpitation, gastrointestinal discomfort, strengthening the stomach, loss of appetite, dysentery and sore swelling	
	Portugal	hyperglycaemia	
*Cosmos bipinnatus*	Brazil	hepatoprotective, headache, jaundice, splenomegaly, stomachaches, flatulence, intermittent malarial fever	[74]
*Forsythia* × *intermedia*	Europe	only ornamental uses stated	[75]
*Gladiolus grandiflorus*		data not found	
*Gomphrena globosa*	India, Bangladesh	heat and empacho, hypertension, antimicrobial, antioxidant, diabetes, hypertension, urinary problems (oliguria), hoarseness, cough, bronchitis and other respiratory diseases, mainly as expectorant, reproductive problems, cytotoxic and estrogenic activity, gangrenous wounds	[48]
	Latin America and Caribbean	jaundice, high cholesterol and urinary problems	
	Other countries	cough, hemorrhage, respiratory, reproductive problems, cancer, pain, antimicrobial, cardiovascular problems, hypoglycemia, urinary and prostate conditions, relaxing nerves and muscles, sedative, antiinflammatory activity	[53,76]
*Hemerocallis disticha*	Taiwan	depression, inflammation, and insomnia	[46]
	Asia	depression, inflammation, and indigestion,	[54]
	Other countries	anodyne, anthelmintic, antidote, antiemetic, antispasmodic, blood purifier, cancer, diuretic, febrifuge, laxative, sedative, aching muscles, and strains, antipyretic, oral disinfectant	[9,11]
*Lonicera* *japonica*	China, East Asia	fever, swelling, dysentery, prolonging life, moisturizing skin and rejuvenation, diuretic, tonic, antipyretic, anti-inflammatory, furuncles, carbuncles, sores and some infectious diseases, chronic enteritis, pneumonia, acute tonsillitis, nephritis, acute mastitis	[60,65,77]
*Paeonia* *lactiflora*	China	irregular menstruation, dysmenorrhea, depression, blood stasis, used for hypochondriac pain, abdominal fullness and distention, improving acuity of vision, protecting skin and beautifying	[67]
	Other countries	cough, haemorrhoids, treatment for varicose veins, aromatherapy	[78,79]
*Pentas* *lanceolata*	Sardinia	anti-malarial, against lung diseases, wound healing	[34]
*Punica* *granatum*	India	conjunctivitis, hematuria, diabetes, anthelmintic, astringent, bactericidal, refrigerant, stimulant, stomachic, hemostatic, alleviating the symptoms of asthma, cardiac conditions, dysentery, diarrhea, dyspepsia, inflammation, hemorrhoids, wounds, ulcers, bruises, sores, mouth lesions, stomatitis, vaginitis, respiratory and urinary tract infections, antipyretic	[51]
	Other countries	throat inflammation, diabetes, obesity, bronchitis, diarrhea, vaginal discharge, pancreas inflammation, cardiovascular disorders, antimicrobial	[61,80,81]
*Robinia* *pseudoacacia*	Slovenia	rheum	[82]
	Italy	sedative	[69]
*Trifolium pratense*	Western countries, China	menopause symptoms, premenstrual syndrome, mastalgia, high cholesterol, and other conditions, women’s health in-medications, to increase resistance to viral and bacterial infections	[52]
	China, Russia	asthma, bronchitis	[47]
	Native American tribes	cancer, pertussis	

### 2.3. Phytochemical Constituents

Polyphenolic compounds (phenolic acids, flavonoids including anthocyanins, tannins and lignans) represent a significant group of natural compounds [83]. These substances, widely distributed in plants (including flowers), are recognized for their protective mechanisms against biotic and abiotic stresses, making them natural antioxidants. When included in human diets, they alleviate oxidative stress at the cellular level, reducing the risk of degenerative, chronic, or civilization-related diseases [84]. Lipophilic carotenoids also play a significant role in mitigating the effects of excess free radicals. These pigment compounds are found in colorful parts of plants, such as flowers (e.g., marigold flower), fruits (e.g., apricots or persimmons) and leaves. Carotenoids play an extensive role in protecting against photooxidative mechanisms and are excellent scavengers of singlet molecular oxygen and peroxyl radicals [85]. Diets rich in carotenoids lower the risk of cancer, cardiovascular diseases, and age-related conditions such as macular degeneration and cataracts [86]. Another group of bioactive plant-derived compounds are terpenoids. This group, which includes monoterpenes, diterpenes, and others, is known for its antimicrobial and anti-inflammatory properties [87]. Compounds with mono- and diterpene structures are also key components of essential oils produced by plants and are among other things stored in flowers of many plant species [88]. The use of plant parts rich in essential oils is well known in phytotherapy worldwide due to their wide spectrum of biological activities, including anti-inflammatory (e.g., *Mentha* sp. [89]), antimicrobial (e.g., *Eucalyptus* sp. [90]), antirheumatic (e.g., Citrus sp. [91]), antioxidant (e.g., *Melaleuca alternifolia* [89]), expectorant (e.g., *Thymus* sp. [92]), sedative (e.g., *Lavandula* sp. [93]), antispasmodic (e.g., *Foeniculum vulgare* [94]), diaphoretic (e.g., *Tagetes minuta* [95]) and diuretic effects (e.g., *Satureja montana* [96]). Studies show that other compounds present in a vast number of herbal medicines are saponins. New research has determined that saponins act as multidirectional compounds and are characterized by anti-inflammatory, antibacterial, antifungal, antiviral, cytotoxic activities, and expectorant effects [97,98]. Saponins are also considered precursors for synthesizing steroidal drugs [99,100]. Additionally, plants are a source of phytosterols. These plant sterols, structurally similar to cholesterol, compete for intestinal absorption, reducing LDL levels and the risk of cardiovascular diseases [101]. Apart from their cholesterol-lowering mechanism, phytosterols can act as anti-diabetic and anti-inflammatory agents [102]. Moreover, fatty acids, including polyunsaturated fatty acids, which are precursors for many important metabolites (e.g., eicosanoids), and amino acids are also synthesized by plants [103,104,105]. Current research focuses on phytochemical diversity and views it as key to discovering new bioactive compounds. The process of obtaining knowledge about new phytochemicals is challenging and complex. However, gaining knowledge about the chemical composition of plants not only supplements existing knowledge but also allows us to infer the direction of biological activity or explain the traditional therapeutic effects known in medicine [106].

The literature describing the chemical composition of selected edible flowers demonstrates the diversity of compounds they contain [107]. Depending on the species, significant differences in phytochemical profiles are observed; however, the most common phytochemicals present in the analyzed edible flowers are compounds belonging to plant polyphenols. The most prominent group includes flavonoids from various subgroups, among which anthocyanins, flavones, flavonols, as well as isoflavonoids (mainly in *T. pratense*), aurones, and chalcones can be distinguished. Phenolic acids, tannins, and lignans also constitute components of some of the described flowers. A detailed phytochemical profile of edible flowers is presented in Table 3.

### 2.4. Biological and Pharmacological Properties

Literature data indicate that various plants’ flowers are used as culinary ingredients and possess biological potential that may be relevant in the treatment of various diseases [45]. The flowers, in both fresh and dried forms, are used as additives in dishes such as salads or beverages, which influences not only the aesthetic and flavor qualities but also enriches the dishes with active compounds, the presence of which has been confirmed in the literature [151]. Given their pharmacological potential, flowers used in cuisine can also be employed as extracts in medicine to support the treatment of various diseases. Furthermore, they serve as a starting point for numerous studies evaluating new, still not fully understood directions of their biological activity [152].

#### 2.4.1. Antioxidant Properties

Antioxidant activity represents a crucial mechanism of action for many plant-derived raw materials [153]. Scientific research suggests that numerous human diseases are linked to the exposure of body cells to oxidative stress from various sources [154]. The ability to neutralize excess free radicals has both preventive and therapeutic significance, often mediated by the presence of compounds with polyphenolic structures [155]. The antioxidant properties of various edible flower extracts have been the subject of extensive scientific investigation. A summary of findings on selected plant species is provided in this section, with detailed results presented in Table 4.

*A. majus* oil exhibits stronger antioxidant activity than extra virgin olive oil, as confirmed by ABTS, DPPH radical, and galvinoxyl radical scavenging assays. Additionally, it protects plasmid pBR322 from H_2_O_2_-induced oxidative damage [156]. Snapdragon (*A. majus*) flowers are rich in aurones, which are particularly effective in combating ROS and reactive nitrogen species (RNS) [157]. These effects are further supported by studies showing reduced iNOS expression associated with water extracts (water extract) [70].

*B. perennis* flowers have demonstrated strong antioxidant potential in vitro. Their ethanolic extract inhibits hydroxyl radicals and nitric oxide production while preventing the formation of thiobarbituric acid-reactive substances [158]. DPPH and ABTS assays, as well as iron-chelating activity, further confirm their efficacy (microwave assisted athenolic extract) [159].

*C. ternatea* flower extracts have shown significant DPPH radical scavenging activity, linked to their high anthocyanin content [160]. Similarly, *C. tinctoria* ethanol extract has good scavenging properties for a number of radicals. These include DPPH, ABTS and hydroxyl radicals. The constituents of tickseed flowers can suppress the expression of nitric oxide in N9 cells induced by lipopolysaccharides [73].

Antioxidant activity has also been observed in in *C. bipinnatus* flowers. It has been shown that DPPH radical scavenging of methanolic extracts depends on the variety and color of the flower [161]. *C. bipinnatus* flower methanolic extract has also been shown to prevent and protect DNA from oxidative damage [162,163].

The flowers of *G. grandiflorus* have high content of anthocyanins, flavonoids and vitamin C which collectively contribute to their high DPPH radical scavenging ability [118]. Similarly, *G. globosa* flowers, containing flavonoids and betacyanins, exhibit promising antioxidative properties [164].

Daylilies (*H. disticha*) also show a strong antioxidative activity. The flower supercritical carbon dioxide extract has the ability to scavenge DPPH, H_2_O_2_, superoxide anion and hydroxyl radicals [127]. Stelladerol, a phytochemical in daylilies, demonstrates potent inhibition of lipid oxidation [165].

Studies indicate the Japanese honeysuckle (*L. japonica*) possesses both natural antioxidant and chemopreventive properties. A recent study has demonstrated that the ethanol extract of this plant exhibited antioxidant activity, as evidenced by its capacity to scavenge DPPH radicals, total reactive oxygen species, hydroxyl radicals and peroxynitrites [65].

*P. lactiflora* (peony) petal flavonoid extracts have been shown to protect BRL3A cells against H_2_O_2_-induced oxidative stress by scavenging reactive oxygen species and activating antioxidant enzyme genes via the Nrf2 signalling pathway [166]. Furthermore, the flavonoid-rich extract has been shown to reduce malondialdehyde levels and enhance the activity of key antioxidant enzymes, including superoxide dismutase and glutathione peroxidase [167].

Hydrophilic extracts of *P. lanceolata* have demonstrated substantial oxygen radical absorption capacity (ORAC), reinforcing their antioxidant potential [12].

Flowers of *P. granatum* (pomegranate) show the ability to reduce superoxide anions and enhance glutathione peroxidase activity, making them promising antioxidants [81]. The methanol–water extract demonstrates strong DPPH radical scavenging and CUPRAC activity, better than Tolox used as reference [143].

The methanolic and ethanolic extracts of *R. pseudoacacia* (black locust) flowers, rich in flavones, exhibit antioxidant activity confirmed through ABTS assays [168].

*T. pratense* (red clover) flowers demonstrate antioxidative properties attributed to their genistein content [169,170]. Recent studies reveal that 50% ethanolic extracts of red clover flowers exhibit cultivar-dependent antioxidant activity, although lower than vitamin C [171].

In contrast, *F.* × *intermedia* flowers do not exhibit significant antioxidant activity. However, other species belonging to the same genus have been subject to study and have been demonstrated to possess this mechanism of action. The flowers of *F. koreana* have been examined in DPPH, superoxide anion, and nitric oxide radical scavenging activity assays. The findings of this study suggest that the flowers of *F. koreana* could potentially serve as a valuable source of antioxidants [172].

**Table 4:** Antioxidant activity of edible flowers.

Plant Species	Type of Assay	Results	Referemce
*Antirrhinum majus*	TPC assay TF assay	TPC = 28.35 ± 1.76 mg GAE/g DW (maceration) TPC = 10.01 ± 0.12 mg GAE/g DW (hydrolisis) TF = 1.8 14 mg CE/g DW	[70]
	DPPH assay	41.7 ± 0.94% (hydrolisis) 32.1 ± 0.98% (maceration)	
	FRAP assay	65.49 ± 0.35 mmol Fe^2+^ equiv./g DW (hydrolisis) 112.63 ± 14.15 mmol Fe^2+^ equiv./100 g DW (maceration)	
*Bellis* *perennis*	TBARS assay ^•^HO assay, NO assays	TBARS = 32.44 ± 4.24% (150 mg/mL ethanol) ^•^HO = 58.85 ± 2.54% (150 mg/mL (ethanol extract) NO = 51.91 ± 2.38% (150 mg/mL (ethanol extract)	[158]
	Obtained phenolic yield DPPH assay, ABTS assay, RP assay ICh (iron chelating)	135.67 mg GAE/g dw DPPH = 46.4 mg GAE/g DW ABTS = 59.6 mg TE/g DW RP = 288.0 mg GAE/g DW ICh = 32.7 mg EDTAE/g DW (microwave-assisted ethanolic extract)	[159]
*Clitoria* *ternatea*	DPPH assay TPC	DPPH IC_50_ = 1 mg/mL (water extract) DPPH IC_50_ = 4 mg/mL (ethanol extract) TPC = 1.9 mg/g extract as gallic acid equivalents	[173]
	DPPH assay FRAP assay H_2_O_2_ assay	DPPH = 62 ± 4.30% (1000 mg concetration) FRAP = 75 ± 5.25% (1000 mg concetration) H_2_O_2_ = 88.9 ± 6.22% (1000 mg concetration) (ethanolic)	[174]
	TPC assay	TPC = 20.7 ± 0.1 mg GAE/g samples (water) TPC = 61.7 ± 0.2 mg GAE/g samples (methanol)	[175]
	DPPH assay	DPPH = 449.33 ± 2.31% (water) (concetration 100 μg/mL) DPPH = 411.33 ± 1.16% (methanol) (concetration 100 μg/mL)	
*Coreopsis tinctoria*	NO assay, Nitrite scavenging asssay, N-nitrosamine formation inhibition assay	NO = SC_50_ = 287.92 ± 7.42 μg/mL (saponins) Nitrate = SC_50_ = 191.63 ± 7.69 μg/mL (saponins) N-nitrosamine = SC_50_ = 1787.4 ± 51.26 μg/mL (saponins)	[73]
	DPPH assay ABTS assay NO radical scavenging assay RC assay	DPPH = IC_50_ = 0.693 mg/mL (polysaccharides) ABTS = IC_50_ = 0.299 mg/mL (polysaccharides) NO IC_50_ = 0.105 mg/mL (polysaccharides) RC = 127.79 ± 2.57 µg Trolox/mg (polysaccharides)	
	DPPH assay ABTS assay radical-scavenging assay a cellular antioxidant activities assay	DPPH IC_50_ < 10 µM (flavonoid compounds) ABTS IC_50_ < 10 µM (flavonoid compounds) IC_50_ < 10 µM (flavonoid compounds) IC_50_ < 10 µM (flavonoid compounds)	
	In vivo/mice/ CCl_4_-induced hepatotoxicity	SOD and GRd activity significantly increased after treatment with 70% ethanol lyophilized extract at doses of 0.5 and 1.0 g/kg. compared CCl_4_ group; GPx activity significantly increased compared CCl_4_ group after treatment with 1.0 g/kg extract	[176]
*Cosmos* *bipinnatus*	TPC assay DPPH assay ABTS assay RP	Methanolic extracts from various colored cosmos flowers: White variety: TPC 361.94 ± 1.45 μM/GAE DPPH IC_50_ = 1.65 ± 0.29 mg/mL ABTS IC_50_ = 1.82 ± 0.17 mg/mL RP IC_50_ = 2.12 ± 0.06 mg/mL	[163]
		Pink variety: TPC 404.37 ± 7.66 μM/GAE DPPH IC_50_ = 1.45 ± 0.31 9 mg/mL ABTS IC_50_ = 1.93 ± 0.48 mg/mL RP IC_50_ = 1.46 ± 0.01 mg/mL	
		Orange variety: TPC 612.21 ± 3.34 μM/GAE DPPH IC_50_ = 0.84 ± 0.08 mg/mL ABTS IC_50_ = 4.27 ± 1.20 mg/mL RP IC_50_ = 1.39 ± 0.07 mg/mL	
		Violet variety: TPC 1012.93 ± 7.86 μM/GAE DPPH IC_50_ = 0.61 ± 0.06 mg/mL ABTS IC_50_ = 1.48 ± 0.07 mg/mL RP IC_50_ = 0.82 ± 0.06 mg/mL	
*Gladiolus* *grandiflorus*	TPC assay TAA assay	TPC 137.1 mg GAEg^−1^FM * TAA = 92.6% (hydroalcoholic extract) * (mean value from different gladiolus varietes: White Friendship, Rose Supreme, Jester, T704)	[118]
*Gomphrena globosa*	DPPH assay	DPPH IC_50_ = 20.35 ± 0.360 μg/mL (crude methanolic extract) DPPH IC_50_ = 13.17 ± 0.308 μg/mL (n-Hexane fraction) DPPH IC_50_ = 20.39 ± 0.245 μg/mL (BHT)	[177]
*Hemerocallis disticha*	Scavenging activity of H_2_O_2_ (chemiluminescence measurement)	EC_50_ = 1.13 ± 0.09 μg/mL	[127]
	Scavenging activity of O^2−^ (chemiluminescence measurement)	EC_50_ = 1.07 ± 0.07 μg/mL	
	Scavenging activity of ^•^OH (chemiluminescence measurement)	EC_50_ = 12.69 ± 1.21 μg/mL	
	DPPH assay	for 0.3–2.7 mg/mL concentration range 7–93% scavenging ability (supercritical carbion dioxide extract)	
	Lipid oxidation inhibition (for stelladerol)	94.6% ± 1.4 at 10 μM	[165]
*Lonicera* *japonica*	DPPH assay total reactive oxygen species hydroxyl radical peroxynitrite radical	DPPH IC_50_ = 4.37 μg/mL IC_50_ = 27.58 ± 0.71 μg/mL IC_50_ = 0.47 ± 0.05 μg/mL IC_50_ = 12.13 ± 0.79 μg/mL (ethyl acetate fraction)	[65]
	TEAC TPC	TEAC = 589.1 μmol Trolox equivalent/100 g DW TPC = 3.63 gallic acid equivalent/100 g DW (methanolic extract, bud)	
*Paeonia lactiflora*	In vivo/SOD activity (flavonoids extract 10–40 mg/kg b.w./day)	significantly increased in a concentration-dependent manner	[167]
	In vivo/CAT activity (flavonoids extract 10–40 mg/kg b.w./day)	significantly increased CAT activity of serum and liver	
	In vivo/GSH-Px activity (flavonoids extract 10–40 mg/kg b.w./day)	significantly increased the GSH-Px activity of serum and liver	
	In vivo/MDA content (flavonoids extract 10–40 mg/kg b.w./day)	significantly reduced the level of MDA	
	DPPH assay	IC_50_ = 4.70 μg/mL (ethyl acetate extract) IC_50_ = 7.31 μg/mL (ethyl ether extract)	[133]
*Pentas* *lanceolata*	ORAC assay TPC assay	HORAC = 130.43 μM TE/g FW LORAC = 0.35 μM TE/g FW TAC = 130.78 μM TE/g FW TPC = 6.52 GAE/g FW (hydrophilic extract; ethanol: water: 0.1% TFA 80:19:1, *v*/*v*/*v*)	[12]
*Punica granatum*	DPPH assay CUPRAC assay	IC_50_ = 0.028 ± 0.004 mg/mL (IC_50 Trolox_ = 0.113 mg/mL) IC_50_ = 0.039± 0.004 mg/mL (IC_50 Trolox_ = 0.064 mg/mL) (pomegrante flower lyophilized extract)	[143]
	In vivo/Lipid peroxidation GSH assays	enhanced by about 100% in the hippocampal brain area of STZ-induced diabetic rats, as evidenced by increased production of the thiobarbituric acid reactive substance, MDA and 4-HDA (*p* < 0.001)	[178]
		STZ + PGF I (300 mg/kg/day ground powder of pomegranate flowers) LPO (MDA + 4-HDA) = 3.41 ± 0.24 GSH = 162.9 ± 3.7 µg/g tissue	
		STZ + PGF II (400 mg/kg/day ground powder of pomegranate flowers) LPO (MDA + 4-HDA) = 3.21 ± 0.24 GSH = 172.8 ± 4.0 µg/g tissue	
		STZ + PGF III (500 mg/kg/day ground powder of pomegranate flowers) LPO (MDA + 4-HDA) = 2.91 ± 0.38 GSH = 179.8 ± 3.3 µg/g tissue	
*Robinia* *pseudoacacia*	TP TNF (total non-flavonoids) TF DPPH ABTS FRAP	TP = 15.96 ± 0.85 (methanolic) mg GAE/g DW TP = 39.58 ± 2.82 (ethanolic) mg GAE/g DW TNF = 10.36 ± 1.47 (methanolic) mg GAE/g DW TNF = 23.63 ± 2.13 (ethanolic) mg GAE/g DW TF = 3.86 ± 0.44 (methanolic) mg CE/g DW TF = 4.53 ± 0.35 (ethanolic) mg CE/g DW DPPH = 24.67 ± 2.93 (methanolic) mg TE/g DW DPPH = 27.92 ± 2.80 (ethanolic) mg TE/g DW ABTS = 17.49 ± 8.14 (methanolic) mg TE/g DW ABTS = 27.79 ± 18.78 (ethanolic) mg TE/g DW FRAP = 7.47 ± 0.38 (methanolic) mg TE/g DW FRAP = 7.38 ± 0.28 (ethanolic) mg TE/g DW	[168]
*Trifolium pratense*	In vivo/assay on adult female Kunming mice (formononetin)	increased activities of superoxide dismutase, glutathione peroxidase, catalase, reduced lipid peroxidation	[179]

ABTS—2,2′-azinobis [3-ethylbenzthiazoline-6-sulfonic acid] diammonium salt; BHT—tert-butyl-1-hydroxytoluene; CAT—cathalase; CE—catechin equivalents; CUPRAC—cupric ion reducing antioxidant capacity; DPPH—2,2-Diphenyl-1-picrylhydrazyl; DW—dry weight; FM—fresh mass; FRAP—ferric reducing antioxidant power; GAE—Gallic acid equivalent; GSH—glutathione; GSH-Px—glutathione peroxidase; LPO—lipid peroxidation; MDA—lipid peroxidation; MDA + HDA4—malondialdehyde + 4-hydroxyalkenals; NO—nitric oxide; ORAC—oxygen radical absorbance capacity; PGF—pomegranate flowers; RC—reducing capacity; RP—reducing power; SOD—superoxide dismutase; STZ—diabetes; TAA—total antioxidant activity; TAC—total antioxidant capacity; TBARS—thiobarbituric acid reactive substances; TE—trolox equivalents; TF—total flavonoid; TP/TPC—total phenolic content.

#### 2.4.2. Neuroactive Properties

Despite significant advancements in medicine, the therapeutic management of nervous system disorders remains a formidable challenge. The demographic shift towards an ageing population has resulted in a concomitant increase in the prevalence of neurodegenerative diseases [180]. Consequently, the prevention and alleviation of neuroinflammatory processes have emerged as one of pivotal objectives in the field of medical research [181]. The exploration of novel sources of substances with high neuroactive potential, particularly within the realm of plant compounds, has emerged as an area of research interest for scientists [182].

The utilization of edible flowers in the culinary traditions of diverse nations, along with their non-toxic characteristics, represents a promising area of research [183]. Recent studies suggest that the bioactive compounds present in these flowers may hold significant therapeutic value in the management of diseases affecting the central nervous system (CNS) [182]. Below, the neuroactive properties of selected edible flowers are discussed, with detailed data presented in Table 5.

Studies have shown that water extracts of Bellis perennis (daisy) exert anxiolytic effects and improve learning performance in animal models [184]. Moreover, Andleeb et al. (2021) showed that the homeopathic preparations of *B. perennis* protect rat pheochromocytoma (PC12) cells by neutralizing the effects of alcohol [185]. The daisy ethanol extract has been shown to positively influence mice with pilocarpine induced seizures. The study suggests the modulatory action on epileptogenesis and promoting anticolvusant and neuroprotective mechanisms [186].

In contrast, Ma et al. (2022) investigated the neuroprotective effect of *C. tinctoria*, employing both a network pharmacology-based method and an in vitro model to validate the outcomes of in silico analyses. The implicated mechanisms include pathways such as cAMP signaling, calcium signaling, HIF-1 signaling, and PI3K-Akt signaling. A research concluded that *C. tinctoria* flower 80% methanolic extract may exert a neuroprotective effect through the BCL-2 and AKT signalling pathways [187]. Moreover, it has been shown that flavanomarein, a predominant compound in the flowers of *C. tinctoria*, demonstrates a significant neuroprotection against 6-OHDA-induced neurodegeneration. This effect is achieved by enhancing the PI3K/AKT signaling pathway while suppressing the NF-κB signaling pathway [188]. Another study found that a 95% ethanol extract of *C. tinctoria* may help alleviate neurodegenerative diseases, possibly through the activation of quinone oxidoreductase 1 (NQO1) and its anti-inflammatory effects on the nervous system [189].

*L. japonica* (Japanese honeysuckle) has shown psychotherapeutic properties in animal models of chronic mild stress. The results of the in vivo tests indicated that the plant material may possess antidepressant and antianxiety effects in chronic mild stress. The observed effects were found to be mediated through the HTR2A/PLCγ/ERK/CREB pathway. Moreover, luteolin, a bioactive compound in *L. japonica,* reversed depressive/anxiety-like behavior in rats exposed to chronic mild stress, with this effect being superior to that of the parallel extract tested [190]. 

The study performed by Cambay et al. (2011) investigated the effects of pomegranate (*P. granatum*) flower supplementation on learning and memory performance in rats with diabetes. The experimental design involved the utilization of the Morris water maze and probe tests to assess cognitive function. The findings demonstrate that pomegranate flower extract successfully restored the initial levels of lipid peroxidation and glutathione in the rats’ hippocampal tissue, leading to a reduction in the increase in glial fibrillary acidic protein (GFAP) levels, which is a recognised biomarker of neurotoxicity [178].

The preclinical studies on red clover phytochemicals suggest them being useful in treating neuroinflammation and cognitive disorders induced by a high-fat diet in mice and Aβ-induced cognitive deficits in rats. Pratensein, an isoflavone, reduced neuroinflammatory markers including malondialdehyde (MDA), nitric oxide (NO), neuronal nitric oxide synthase (nNOS), IL-1β and TNF-α. Additionally, it decreased Aβ levels and activity of acetylcholinesterase, but increased the expressions of synapse plasticity-related proteins, cAMP-response element binding protein (CREB), the brain derived neurotrophic factor (BDNF), Ca^2+^/calmodulin kinase II (CaMKII), N-methyl-D-aspartate receptor 1 (NMDAR1), postsynaptic density protein 95 (PSD-95), protein kinase A beta subunit (PKACβ) and protein kinase C gamma (PKCγ). Pratensein enhances synaptic plasticity and increases cholinesterase activity in the brain [191].

Another isoflavone formononetin improved cognitive function in mice with high-fat diet-induced cognitive decline. It is achieved by inhibition of neuroinflammation regulated by the PGC-1α pathway [192].

**Table 5:** Neuroactivity of edible flowers.

Plant Species	Type of Assay	Model	Results	Reference
*Bellis perennis*	In vivo/Wistar rats 20 and 60 mg/kg of H_2_O extract of flowers	Open field and elevated plus maze tests (anxiety-like behavior)	Open field—spent more time at the center, showed less mobility and velocity Elevated plus maze—spent more time in the open arms, spent less time in the closed arms, were less mobile, were slower and rotated less frequently	[184]
		Morris water maze (spatial memory)	Spent more of the time to find the platform	[184]
	In vitro	PC12 cell line/MTT	*B. perennis* (homeopathic potent: 6C and 30C) treatment at concentration of 2 µL/mL, 4 µL/mL and 8 µL/mL significantly increased % cell viability (97.06%, 90.62%, 99.71% and 99.87%, 93.40%, 87.35% respectively)	[185]
*Coreopsis* *tinctoria*	In vitro 80% methanol extract	SH-SY5Y cell line	Extract (150 μg/mL) remarkably reversed neuronal apoptosis, which was induced by MPTP	[187]
			pretreatment of the extract significantly attenuated MPTP-induced ROS level (3.24-fold of that in the control at 150 μg/mL)	
			Pretreatment of the extract improved the protein level of BDNF in MPTP-induced cells	
			Extract (150 μg/mL) regulated gene and protein expression (caspase-3, BCL2, BAX, AKT, PI3K, and HIF-1)	
*Lonicera japonica*	In vivo	chronic mild stress model/rats	A notable antidepressant response of flowers (100 mg/kg/day of *L. japonicae* flos)	[190]
		Elevated plus-maze test/rats	Significantly increases the number of entries in the open arms (100 mg/kg/day of *L. japonicae* flos)	
		Forced swim test/rats	Significant reduction in the immobility time (100 mg/kg/day of *L. japonicae* flos)	
		Neurotransmitter release in rat striatum/rats	*L. japonica* flower can improve release of neurotransmitters (5-HT, 5-HIAA, DA, DOPAC and HVA) (100 mg/kg/day of *L. japonicae* flos)	
*Punica granatum*	In vivo	Morris water maze and probe tests/STZ diabetic rats	Supplementation of pomegranate flowers led to improvements in learning and memory performances of diabetic rats (300–500 mg/kg/day of powdered flower mixed at 1.5, 2 or 2.5% with commercially available rat feed)	[178]
*Trifolium pratense*	Randomized, controlled clinical trial	Cognitive function in postmenopausal women (>60 years)	Appeared to not have major short-term effects on cognitive function (2 tablets/6 months; 1 tablet: formononetin 25 mg, biochanin 2.5 mg and less than 1 mg of daidzein and genistein)	[193]

BDNF—brain-derived neurotrophic factor; MPTP—1-methyl-4-phenyl-1,2,3,6-tetrahydropyridine; PC12—rat pheochromocytoma; SH-SY5Y is a human derived cell line often used as in vitro models of neuronal function.

#### 2.4.3. Anti-Inflammatory Properties

Inflammation plays a critical role in the development of numerous diseases and disorders of the body [194]. Consequently, anti-inflammatory agents represent one of the most frequently employed groups of drugs in therapeutic interventions [195]. There is growing scientific interest in the use of plant-based bioactive compounds, and flowers classified as edible have emerged as promising candidates for mitigating inflammatory responses [196,197] (Table 6).

Numerous studies report that extracts from edible flowers demonstrate significant anti-inflammatory properties through diverse mechanisms. *B. perennis* extracts prepared using methanol, dichloromethane, hexane and water were evaluated by their ability to inhibit NO production in LPS-stimulated RAW 264.7 macrophages. They showed high anti-inflammatory potential [71]. 

Similarly, petroleum ether extracts of *C. ternatea* flowers demonstrated anti-inflammatory and analgesic effect, particularly at the highest doses administered [111].

A number of studies explore the molecular mechanisms underlying the anti-inflammatory effects of edible flowers. Hou et al. (2017) examined the effects of flavanomarein, a significant flavonoid from *C. tinctoria*, on the viability of hypoxic HK-2 cells. The study revealed that the compound led to a reduction in oxidative stress, pro-apoptotic factors, and notably pro-inflammatory factors, as well as the activation of NF-κB in TECs under conditions of hypoxia/reperfusion (H/R) [73].

Michalak et al. (2018) investigated the anti-inflammatory profile of 75% methanol extract of *F.* × *intermedia* flowers, identified as a valuable source of bioactive lignans. The incubation of LPS-stimulated neutrophils with this extract (25–100 μg/mL) results in a statistically significant reduction in the release of IL-8 and TNF-α and an inhibitory effect on integrin surface expression. However, the preincubation of neutrophils with extract did not prevent neutrophil attachment to endothelial cells [75].

In the study undertaken by Sherif (2021), the anti-inflammatory activity of *G. globosa* was investigated. The extracts (water, hydroalcoholic) derived from the flower above have a reducing ability, an ability to inhibit albumin denaturation, a capacity to stabilize red blood cell membranes, and an anti-proteinase action [53]. 

Research on *L. japonica* offers a more comprehensive exploration of both in vitro and in vivo models. Studies using A549 cells reveal that n-butanol fraction inhibited the COX-1 and COX-2 activity, as well as the expression of IL-1-induced COX-2 protein and mRNA [65]. The findings are supported by carrageenan/formaldehyde-induced rat paw swelling, mouse ear oedema, cotton ball granulomatous hyperplasia, mouse cutaneous vascular hyperpermeability and egg white-induced localized acute inflammation [65]. Furthermore, the water extract also exhibits anti-inflammatory effect in rat cervicitis model, *Escherichia coli*-infected mouse model, excision wound model of an infected rat and ovalbumin-induced rat asthma model. The mechanisms include inhibition of inflammatory factor synthesis/release, decreased expression of immune related molecules, and enzyme activities of matrix metalloproteinase [198]. In an in vivo rat model, it was demonstrated that *L. japonica* flower aqueous extract reduced TNF-, IL-1 and IL-6 levels in both serum and bronchoalveolar lavage fluid, decreased in the levels of oxidative stress factors (MDA and MPO), and increased in the activities of SOD and GSH-Px in the lung tissue [199]. 

*P. lactiflora* flowers have demonstrated anti-inflammatory efficacy in in vitro and in vivo. Extracts from *P. lactiflora* modulate cytokine mRNA expression and/or cell signalling protein expression [200]. Peony ethanolic extract has been shown to possess the ability to inhibit the release of NO and inflammatory factors. Furthermore, it has been demonstrated to inhibit the activation of the MAPK and NF-κB signalling pathways in cells, which may represent an additional significant mechanism in the anti-inflammatory process [201]. Further mechanisms of action include potent antiglycation capacity, significant inhibition of tyrosinase, COX-2, and collagenase (water and athanol extracts) [67]. 

In turn, *P. granatum* ethanolic and methanolic extracts have been shown to possess anti-inflammatory properties, as evidenced by studies conducted using albumin denaturation, membrane stabilization, anti-proteinase and anti-lipoxygenase assays [202]. The ethanolic extract modulated the activity of various mediators and cytokines that play a pivotal role in the inflammatory process. Their ability to inhibit the phosphorylation of ERK1/2, p38, JNK and the translocation of the NF-B p65 subunit into nuclei was reported [203]. Furthermore, the anti-inflammatory properties of the hydroalcocholic extract from pomegranate flowers were substantiated by Gheith and El-Mahmoudy (2017) through the implementation of a human red blood cell-membrane stability test. This is due to the fact that the extract exhibited a protective effect on erythrocyte membranes against lysis [204]. 

Finally, the anthocyanin-rich extract from *T. pratense* hasdemonstrated significant inhibition of LPS-induced inflammation and oxidation in LPS-treated RAW 264.7 macrophages in vitro [205].

**Table 6:** Anti-inflammatory activity of edible flowers.

Plant Species	Type of Assay	Model	Results	Reference
*Bellis perennis*	In vitro	RAW 264.7 macrophage cells stimulated by LPS	Inhibitory effect on LPS-induced NO secretion extracts (20 µg/mL): methanol (96.3% inhibition) dichloromethane (86.4% inhibition) hexane (25.3% inhibition) water (14.2% inhibition)	[71]
*Clitoria* *ternatea*	In vivo	Carrageenan rat paw edema model	The petroleum ether (60–80 °C) extract possessed significant anti-inflammatory properties (400 mg/kg)	[111]
		hot-plate assay in mice model	The petroleum ether (60–80 °C) extract possessed significant analgesic properties (400 mg/kg)	
	In vivo	ICR mice/CCl4 -induced hepatotoxicity	Significantly reduced the levels of TNF-α (60.82 pg/mg protein) and IL-1β (1168.69 pg/mg protein) by 0.5 and 1.0 g/kg of 70% lyophilized ethanol extract compared to CCl_4_ level (82.74, and 1608.68 pg/mg protein, respectively)	[176]
*Coreopsis* *tinctoria*	In vitro	BV-2 cell stimulated by LPS	Significant inhibitory effect on NO production induced by LPS (ethyl acetate extract) LPS-induced upregulation of mRNA expression levels for iNOS, COX-2, IL-1β, IL-6, and TNF-α.	[206]
*Forsythia* × *intermedia*	In vitro	LPS-stimulated neutrophils and monocytes/macrophages	Incubation for 24 h; (25–100 µg/mL): significant reduction of IL-8 and TNF-α release At the highest concentration, decreased the surface expression of CD11a/CD18	[75]
	In vitro	Attachment of LPS-stimulated neutrophils to the TNFα-stimulated endothelium	Dual preincubation of neutrophils (12.5; 25; 50 µg/mL) and HUVEC (12.5; 25; 50 µg/mL) with extracts: significant reductions in adherence (lyophilized methanol extract)	
*Gomphrena globosa*	In vitro	Inhibition of albumin denaturation	87.71% of inhibition (decoction) 80.70% of inhibition (hydroalcoholic extract) compared to 91.22% (diclofenac sodium) concentration: 500 g/mL	[53]
		Anti-proteinase activity	about 90% of inhibition (decoction) 87.71% of inhibition (hydroalcoholic extract) concentration: 500 g/mL	
*Lonicera japonica*	In vivo/mice and rats models:	AA ear edema, croton-oil ear edema, CGN-paw edema, rat cotton pellet granulomatic AIA-inflammation	n-butanol fraction *L. japonica*; inhibition: 27%, 23%, 26%, 18% and 42%, respectively to the models (oral doses of 400 mg/kg) compared to 27%, 13%, 13%, 0% and 58% (aspirin at 100 mg/kg)	[65]
*Paeonia lactiflora*	In vitro	COX-2 inhibition	IC_50_ = 10.06 ± 0.82 μ g/mL (ethanol) IC_50_ = 11.95 ± 0.97 μg/mL (water)	[67]
		Collagenase inhibition	IC_50_ = 262.6 ± 15.7 μg/mL (ethanol) IC_50_ = 144.5 ± 12.8 μg/mL (water)	
		monophenolase assay	IC_50_ = 189.8 ± 10.2 μg/mL (ethanol) IC_50_ = 232.9 ± 4.4 μg/mL (water)	
		Diphenolase assay	IC_50_ = 72.1 ± 4.8 μg/mL (ethanol) IC_50_ = 89.2 ± 4.2 μg/mL (water)	
*Punica granatum*	In vitro	Inhibition of albumin denaturation	IC_50_ = 214 μg/mL (methanolic) IC_50_ = 309 μg/mL (aqueous) Aspirin inhibition: 75.66 ± 0.4% (100 μg/mL)	[202]
		Anti-proteinase activity	IC_50_ = 305 μg/mL (methanolic) IC_50_ = 352 μg/mL (aqueous) Indomethacin inhibition: 80.20 ± 0.12% (100 μg/mL)	
		Anti-lipoxygenase activity	IC_50_ = 328 μg/mL (methanolic) IC_50_ = 375 μg/mL (aqueous) Indomethacin inhibition: 64.63 ± 0.28% (100 μg/mL)	
		RAW 264.7 macrophage cells stimulated by LPS	ethanolic extract: NO production reduced (IC_50_ = 31.8 μg/mL) PGE_2_ production reduced (IC_50_ = 54.5 μg/mL) IL-6 production reduced (IC_50_ = 48.7 μg/mL) IL-1β production reduced (IC_50_ = 71.3 μg/mL) TNF-α production reduced (IC_50_ = 62.5 μg/mL)	[203]
		Human red blood cell-membrane stability	Hemolysis IC_50_ = 126.1 ± 1.35 µg/mL (50% hydromethanolic)	[204]
*Trifolium pratense*	In vitro	RAW 264.7 macrophage cells stimulated by LPS	Reduction in the expression of pro-inflammatory genes: TNFα, IL1β, and iNOS (RC at 5 and 10 µg/mL, 80% methanolic extract) Downregulation of MCP1 (up to 64.4% ± 1.1%) and COX-2 (up to 39.9% ± 2.6%) expression (RC at 5 µg/mL 80% methanolic extract) Suppression of IL1β, iNOS, MCP1, and COX2 gene expression (RCA at 5 µg/mL)	[205]
		TNFα secretion	Inhibition of TNFα secretion in a dose-dependent manner (10%, 15%, and 25% at 5, 10, and 20 µg/mL, respectively)	

AA—arachidonic acid; AIA—adjuvant-induced arthritis; CGN—carrageenan; COX-2—cyclooxygenase-2; LPS—lipopolysaccharide; NO—nitric oxide; RC—red clover extract; RCA—red clover anthocyanins fraction; HUVEC—human umbilical vein endothelial cell.

#### 2.4.4. Other Important Pharmacological Properties

##### Anti-Microbial Properties

*A. majus* extracts have been shown to possess significant antimicrobial properties against a diverse range of bacterial and fungal strains [56]. These include bacterial strains such as *Staphylococcus aureus*, *Bacillus subtilis*, *Pasteurella multocida* and *Escherichia coli*, as well as fungal strains including *Rhizoctonia solani* and *Aspergillus niger*. The antimicrobial potential of *A. majus* methanolic extract was evaluated using the disc diffusion method [156]. Furthermore, ethanolic extracts exhibited activity against *Bacillus cereus*, *Bacillus brevis*, *Staphylococcus epidemitus*, *Micrococcus luteus*, *Mycobacterium phlei* and *Bordetella bronchiseptica* [207]. Furthermore, the microtiter-plate dilution assay revealed the antimicrobial activity against *Bacillus subtilis*, *S. aureus*, *Enterobacter aerogenes*, *E. coli*, *Candida albicans* (80% methanol extract) [157].

The *B. perennis* extract demonstrated a broad-spectrum antibacterial effect against Gram-positive bacteria such as *Streptococcus pyogenes*, *S. aureus* and *S. epidermidis*, as well as the Gram-negative bacteria *Enterobacter cloacae* [98]. Another study reported moderate activity against *S. epidermidis* MU30, *P. aeruginosa* ATCC 27853 and *P. fluorescens* MU 181. Additionally, water extract demonstrated promising antiquorum-sensing activity on *Chromobacterium violaceum* CV026 [208].

Various extracts from butterfly pea (*C. ternatea*) have exhibited inhibitory effects against multiple bacterial pathogens, including *Pseudomonas aeruginosa*, *E. coli*, *Klebsiella pneumonia*, *B. subtilis*, *Aeromonas formicans*, *Aeromonas hydrophila* and *Streptococcus agalactiae*. Beyond antibacterial properties, *C. ternatea* also possesses antiparasitic and insecticidal effects. It induces paralysis within 15–20 min and brings death within 28–30 min to the Indian earthworm (*Pheritima posthuman*) [111].

Globe amaranth (*G. globosa*) has shown antibacterial activity against *E. coli* and *S. aureus* [209].

Essential oil and extracts from *L. japonica* flowers have exhibited a remarkable action against *Listeria monocytogenes*, *B. subtilis*, *B. cereus*, *S. aureus*. The agar-well diffusion in vitro method also showed antibacterial activity of flower buds extract against *B. cereus* and *S. aureus* [65].

Both in vivo and in vitro assays have validated the bacteriostatic and antibacterial properties of honeysuckle flowers. They exhibit a broad antimicrobial spectrum, potent antibacterial activity, and efficacy against drug-resistant bacteria. The strongest action of water and ethanolic extracts has been observed against pathogens such as *P. aeruginosa*, *B. subtilis*, *S. aureus*, *C. albicans*, *E. coli*, *Salmonella Typhi*, *Shigella dysenteriae* and *P. aeruginosa* [198]. In addition to antimicrobial properties, Japanese honeysuckle also shows antiviral activity tested against the respiratory syncytial virus (RSV) using the cytopathologic effect (CPE) assay. The honeysuckle aqueous extract has demonstrated therapeutic potential in a mouse model of influenza A virus-induced pneumonia [210]. Compounds found in honeysuckle flowers inhibit various viruses, including *herpes simplex* keratitis, influenza virus pneumonia, influenza A virus, porcine reproductive and respiratory syndrome virus, Newcastle disease virus, respiratory syncytial virus, influenza virus, and human cytomegalovirus. It also significantly inhibits cytomegalovirus in guinea pigs, pseudorabies virus strain MinA, influenza virus variant FM1, Coxsackie *β*3 virus, enteric cytopathic human orphan 19 virus. These effects are achieved by enhancing drug binding to ceramidase, thereby increasing both antiviral potency and organ protection in cases of influenza [199]. 

Phytochemicals from *P. lactiflora* (peony) have shown the ability to inhibit the synthesis and replication of several viruses, primarily by targeting viral protease and/or polymerase activity. Research has demonstrated antibacterial and antifungal mechanisms involving cell wall modifications that restrict microbial growth and increase susceptibility to phagocytosis [200].

*P. granatum* flowers are effective sources of compounds in treatment of *Salmonella enteriditis* and *Salmonella kentucky* [81].

Lastly, methanoic and ethanolic extracts of *R. pseudoacacia* exhibit antimicrobial activity against pathogens such as *P. aeruginosa*, *B. cereus* and *C. albicans*, *Candida parapsilosis* [168].

##### Anti-Cancerous Properties

Numerous studies have demonstrated the anti-cancerous potential of flower extracts and their phytochemicals. For instance, *B. perennis* flower extracts exhibit antiproliferative activity against human breast cancer cell line (MCF-7) and hepatocellular carcinoma (HepG2/C3A) cancer cells [211]. Furthermore, the oleanane-type triterpene saponins derived from common daisy flowers have shown inhibitory effects on the proliferation of human digestive tract carcinoma cell lines, including HSC-2, HSC-4 and MKN-45 [212].

*C. ternatea* flower aqueous and methanolic extracts have been evaluated across various cancer cell lines including hormone-dependent breast cancer cells (MCF-7), a non-hormone-dependent breast cancer cell line (MDA-MB-231), a human ovary cancer cell line (Caov3), a human cervical cancer cell line (Hela), a human liver cancer cell line (HepG2) and a human foreskin fibroblast cell line (Hs27). The most significant outcomes were observed against MCF-7 cells [111].

The antitumor activity of *C. tinctoria* total flavonoids was evaluated in a study on lung cancer. The mechanism of action may be related to the inhibition of the PI3K-Akt signaling pathway and the activation of the mitochondrial-mediated apoptosis pathway [213]. Additionally, tickseed flower extracts have shown anti-leukemia effects in vitro, highlighting their potential as drug candidates for leukemia treatment [214]. *C. tinctoria* also inhibits the enzymatic activity of aromatase which is an important factor in anti-cancerous activity [215].

The phytochemicals from the Chinese peony (*P. lactiflora*) have been shown to regulate apoptosis by modulating the activity of the key enzymes, caspases-3 and -9, which are pivotal components of the signalling pathway. The isolated compounds have been observed to impede cell proliferation and, in addition, to render cancerous cells susceptible to the effects of chemotherapy agents. Beyond this, these phytochemicals have been demonstrated to exert an influence on the processes of angiogenesis and metastasis. One of the studied cell lines was HT29 colorectal cancer cells [200].

Pomegranate flower (*P. granatum*) 80% ethanolic extract has been shown to exert an inhibitory effect on the proliferation of human breast cancer cells, MCF-7 [61].

*R. pseudoacacia* flower extract (50% ethanolic) has been shown to induce significant necrosis and apoptosis of the HeLa cell, confirming its antitumor effects in vitro [216].

Genistein, an isoflavone of *T. pratense*, exhibits an inhibitory effect on protein tyrosinase kinase and angiogenesis, which may be associated with its antioxidative properties [169]. Red clover has been found to exhibit a significant inhibitory effect on adenosine deaminase in gastric cancer cells resected during surgical operation. However, it did not demonstrate any influence on the enzyme in non-cancerous cells [150].

##### Wound Healing, Antidiabetic, and Organ-Protective Effect

An in vivo study on Wistar albino rats revealed the wound healing capability of *A. majus* to be the most effective among other tested substances [157]. A series of tests were performed to determine the wound healing capability of A. majus flowers, including visual examination, wound-length measurement and estimation of hydroxyproline content. The study attributed this efficacy to the presence of β-sitosterol and tocopherols, which are considered to be among the primary constituents of *A. majus* flowers [157].

The wound-healing properties of *B. perennis* flowers have been also studied. The addition of an extract to the treatment increased the collagen production, epidermis thickness, fibroblast distribution and wound contraction ratio. A study revealed increased levels of Bcl2-mRNA and reduced levels of Bax and p53. The upregulation of these genes has been demonstrated to accelerate wound healing, particularly during the proliferative phase [217]. In addition to the wound-healing properties, daisy flowers have been shown to protect keratinocytes from UVA exposure, with the extract demonstrating a significant reduction in cleaved caspase-3 levels and protection against reactive oxygen species accumulation in keratinocytes. It has been demonstrated that the activity of catalase is increased, whilst glutathione peroxidase activity and IL-6 production enhanced by UVA are reduced [217]. Common daisy aqueous flower extract can reduce the probable cause of hematotoxicity and nephrotoxicity when tested in mice. This experiment suggests that *B. perennis* flowers have protective properties on the kidneys and blood [218]. *B. perennis* flowers possess osteogenic differentiation potential (differentiation of C3H10T1/2 into osteoblasts). This was determined by measuring alkaline phosphatase activity, alizarin red staining and the expression of osteocalcin (immunostaining and qRT-PCR assays) differentiation of C3H10T1/2 into osteoblasts [219]. Further study was conducted that revealed the common daisy to be capable of activating growth factors, bone regeneration genes and proteins for osteoblasts [220]. Research has determined the antidiabetic properties of *B. perennis*, with flower ethanolic extracts having the capacity to reduce blood glucose levels. It is therefore considered a suitable candidate as a food supplement for the prevention and additional therapy of T2DM. Furthermore, common daisy extracts have been shown to induce GLUT4 translocation in in vitro cell systems [221].

The *C. ternatea* aqueous flower extract has been studied in alloxan-induced diabetes in rats assay to determine its antidiabetic affect. The study revealed that the flowers reduced serum glucose, glycosylated hemoglobin and the activities of gluconeogenic enzyme, glucose-6-phosphatase. However, the study also found that the flowers increased serum insulin, liver and skeletal muscle glycogen and the activity of the glycolytic enzyme, glucokinase [111]. Butterfly pea has also proven to have an antihyperlipidemic effect [160].

The potential health benefits of *Coreopsis tinctoria* have been a subject of considerable interest. Studies have demonstrated that tea made from the flowers exhibits hypoglycemic effects, attributed to the inhibition of α-glucosidase. Furthermore, the tea has been observed to enhance insulin sensitivity in rats fed a high-fat diet, and even prevent hepatic metabolic disorders. A further study determined that *C. tinctoria* flowers can protect the kidneys in the early stage of diabetic kidney disease by activating phosphorylation of AMPKα [222]. The flavone content of tickseed flowers has been demonstrated to reduce blood pressure in hypertensive rats or mice models, including those induced by high salt, cold shock, renal, spontaneous and other factors. Compounds derived from tickseed flowers have also been shown to possess angiotensin I-converting enzyme (ACE) inhibitory activities [73]. In addition, tickseed flower attenuated the injury of the kidney in vivo, downregulated blood urea nitrogen, serum creatinine, and urinary albumin, and upregulated creatinine clearance [73]. Treatment with *C. tinctoria* has been shown to have a hepatoprotective effect against carbon tetrachloride-induced liver damage in the murine model. Consequently, the levels of ALT and AST have significantly decreased [176]. The antihypertensive action in spontaneously hypertensive rats is attributable to the decreased mRNA expression of ACE, AT1R and TGF-β1 in the left ventricle, as well as the increased ACE II [223]. Tickseed also acts as a vasodilator [224].

The *G. globosa* flower extract can ameliorate levels of cardiac enzymes, increase levels of HDL and decrease the lipid profile [225]. Moreover, it can reduce LDL in the body [164].

Flavones from *L. japonica* have a hepatoprotective effect. It was determined on immunological liver injury in mice. In addition, honeysuckle has been shown to possess the ability to inhibit the increase in blood sugar levels, reduce serum cholesterol levels, and decrease the accumulation index of atherosclerosis in mice induced by alloxan. Organic acid compounds from *L. japonica* possess antithrombotic effects on the oxidative injury of human umbilical vein endothelial cells [65]. The study on the dry yeast-induced rat fever model and the IL-1*β*-induced fever model in New Zealand rabbits determined honeysuckle flowers to have antipyretic effects. This may be attributable to the expression of prostaglandin E2 receptor EP3 at the preoptic area of hypothalamus (POAH) neurons. The flower extract from *L. japonica* acts protectively against alcohol-induced chemical liver injury in mice and acute liver injury induced by intraperitoneal injection of carbon tetrachloride in mice [198].

*P. lactiflora* constituents present the capacity to modulate blood glucose by affecting glucose uptake and insulin release. In addition to this, these compounds have been shown to exert an indirect effect on the regulation of carbohydrate absorption and metabolism. Some studies suggest that there is potential in modifying damage caused by glycaemic disease [200]. Furthermore, an additional assay determined the ability of peony extract to increase cell viability under UVA exposure (CCD-986Sk fibroblast cell line) [207].

*P. granatum* flowers are known for their antidiabetic activity. The flowers can significantly lower the blood glucose level in cases of type II diabetes through a variety of mechanisms. According to studies, the administration of pomegranate flower extract can result in reduction in fasting blood glucose, total cholesterol, triglycerides, low-density-lipoprotein cholesterol, very-low-density-lipoprotein cholesterol and tissue lipid peroxidation. Concurrently, it has been observed that pomegranate flower extract has the capacity to elevate high-density lipoprotein. Recent studies show that *P. granatum* flowers might prevent diabetic sequelae by peroxisome proliferator-activated receptor-gamma binding and nitric oxide production [140]. Further study established that pomegranate flower extracts have the capacity to prevent type II diabetes by reducing the blood glucose levels and, in turn, inhibiting the α-glucosidase enzyme by increasing postprandial hyperglycemia in type II patients [81]. Subsequent studies suggest that Peroxisome Proliferator-Activated Receptor gamma (PPAR-γ) may serve as a potential molecular target for pomegranate flower extract in anti-diabetic action [226]. In addition, a study conducted on rats demonstrated that pomegranate flower extract enhances the healing of burn wounds (study on rats) very well in comparison to normally used preparations [227]. Another assay in an animal model revealed that *P. granatum* flower extract lowers intraperitoneal adhesion after abdominal surgeries [228]. 

It has been reported that *T. pratense* constituents (isoflavones) may reduce pulmonary hypertension syndrome incidence and improve pulmonary vascular remodelling in animal models. Furthermore, new studies showed the influence of flower phytochemicals on blood components [150].

### 2.5. Critical Analysis and Research Outlook

The literature reviewed shows that edible flowers have a variety of pharmacological properties and could be used for therapeutic purposes. However, there are several important issues that need to be addressed.

Edible flowers exhibit a wide range of bioactivities, including antioxidant, neuroactive and anti-inflammatory, the potential of which varies according to the species tested, as well as the type of extract tested. While the antioxidant activity of the tested edible flowers was characterised for all selected species, for neuroactive and anti-inflammatory activities the data were less extensive and did not include flowers of *H. disticha* and *P. lanceolata*. Interestingly, flowers of *F.* × *interemedia* had lower antioxidant activity than others. Furthermore, flowers such as *A. majus* and *B. perennis* exhibit potent antimicrobial properties against several bacterial and fungal strains, while extracts of *C. tinctoria* and *P. granatum* show potential in inhibiting cancer cell proliferation and modulating key cellular pathways. In addition, *P. granatum* and *C. tinctoria* flowers offer anti-diabetic and cardioprotective effects, supported by mechanisms such as α-glucosidase inhibition and improved insulin sensitivity.

Even though we reached some conclusions, it is important to remember that it is hard to compare the biological activity seen in early studies because different research teams used different methods. This can lead to differences in the results for both the samples and the standards. The reason for this is that the research methods are not standardized. For example, there might be differences in how the extracts are prepared and in the experimental designs. This makes it difficult to understand the results, which are used to select the most active extracts.

While the described biological properties of edible flowers demonstrate promising therapeutic potential, it is essential to note that the majority of these findings are based on preclinical models (in vivo and in vitro). This limits the possibility of application in therapeutic or industrial contexts. Further studies should therefore be carried out, particularly in clinical models, to confirm the efficacy observed in preclinical studies, to confirm the dosage and to allow the assessment of potential side effects in humans.

In addition, the available work does not focus on explaining the synergistic effect of extract components. It is evident that plants constitute a complex chemical matrix. Consequently, the biological activity of edible flower extracts may be attributed to the synergistic interaction of individual chemical compounds. Therefore, studies are needed to determine which compounds or their combinations are responsible for the demonstrated biological activity.

### 2.6. Limitations, and Implications for Human Applications

The results presented in this study mainly concern the biological activity and pharmacological potential of edible flowers in the form of extracts. Due to the limited clinical data available on this topic, the usefulness of these plant products in nutrition and medicine was presented regarding the results of pre-clinical studies. However, it is important to note that although in vitro studies are undeniably valuable research tools, allowing species-specific, simpler, more convenient, and more detailed analysis than whole-organism studies, the results obtained in vitro do not fully reflect the complexity of processes occurring in a living organism. As such, in vitro experiments often serve as a preliminary step before conducting in vivo studies.

It is important to emphasize that overinterpreting results obtained in vitro and directly extrapolating them to living organisms, including humans, may lead to misuse and overstatement [229]. Literature data clearly indicate that most potential drugs that show efficacy in vitro prove ineffective in vivo due to issues related to drug delivery to the affected tissues and toxicity to essential organs [230,231]. Therefore, in vitro results, including IC_50_ values, generally cannot be directly extrapolated to suggest reactions of the entire organism in vivo. Moreover, translating these doses to humans presents challenges due to interspecies differences, differences in metabolism, and pharmacokinetics. Animal models also have their limitations, and inter-species differences undermine the reliability of extrapolating preclinical model results to humans [232].

## 3. Materials and Methods

Relevant studies were identified using electronic databases including PubMed, Google Scholar, ResearchGate, Cochrane, and Elsevier. We also examined bibliographies of selected articles to identify additional sources. The literature search was conducted by combining botanical names, common names, and the specific plant part (flowers) with keywords such as “neuroprotective”, “toxicity”, “culture”, “traditional use”, “(edible) flowers”, “functional flowers”, “neuroprotective potential”, “phytotherapy”, “infusion”, “extract”, “ethnobotany”, “physiological effect”, “nutritional and medicinal properties”, “neurotoxicity”, and “neuroactive”. Based on the obtained search results regarding biological activity, including in vitro and in vivo experiments, pharmacological research, and some clinical studies, the selection of articles was assessed to determine the most relevant sources.

Inclusion criteria encompassed reports, books/book chapters, journal articles, and web pages explicitly mentioning the researched plant part (flower) and providing relevant information about their biological activity or traditional use. Articles were excluded if the full text was not available, the plant part of interest was not mentioned, or the content fell outside the scope of this review. Review articles were generally excluded unless they provided critical context when original research papers were unavailable.

## 4. Conclusions

The review shows that edible flowers have important pharmacological potential. It reveals that the edible flower species exhibit antioxidant properties by demonstrating radical scavenging capacity. Furthermore, selected species are shown to possess neuroactive potential, protecting against the neurodegenerative processes and cognitive disorders that are mediated by specific pathways. In addition to these properties, edible flowers are also observed to exhibit anti-inflammatory activity. Furthermore, studies of extracts derived from the 15 edible flowers tested demonstrate antimicrobial, anticancer, antidiabetic and skin-healing properties. Numerous in vitro studies underscore the potential of edible flowers; however, further research is necessary to expand this knowledge beyond animal models to clinical trial models.

## Figures and Tables

**Table 1:** List of reviewed pants with botanical families they belong.

Family	Plant Name	Common Name	Plant and Flower Description	The Family Characteristic	References
*Asteraceae*	*Bellis perennis* L.	common daisy	Native to Europe; a perennial herb thriving in temperate regions; the plant’s inflorescence consisting of bearing ray and disk flowers; blooms from December to September.	Over 32,000 species; the flower composed of ray flowers and disc flowers. Ray flowers with strap-shaped petals, while disc flowers are tubular with fused petals.	[16,17]
*Asteraceae*	*Coreopsis* *tinctoria* Nutt.	plain coreopsis, garden tickseed	Native to the northwestern and eastern United States; an annual plant producing many flower heads in open, leafy bracted clusters, with distinctive ray flowers in shades of red and orange. It blooms from June to September.	Please see above.	[18]
*Asteraceae*	*Cosmos* *bipinnatus* Cav.	garden cosmos	Native to tropical America; an annual plant with inflorescences having long peduncles; the flower heads with ray flowers in shades of white, pink, or purple, and disk flowers. It blooms from April to August.	Please see above.	[19]
*Amaranthaceae*	*Gomphrena globosa* L.	globe-amaranth	Native from Brazil, Panama and Guatemala, spread to other continents (e.g., Europe); produces purple clover-like flower heads.	With about 2000 species; growing in tropical to cool temperate regions; annuals, perennials, subshrubs, shrubs, lianas or trees. Flowers mostly actinomorphic; inflorescences (often 3-flowered) include spikes, racemes, or panicles.	[20,21,22,23]
*Asphodelaceae*	*Hemerocallis* *disticha* Donn	daylily	Found in Southeast Asia, including China, Japan, and Korea (temperate biome); growing as a perennial with solitary orange, red-orange, or pink flowers.	About 740, in the Mediterranean, Africa, Asia, and Australia; often succulent herbs or pachycaul trees; flowers are petaloid, regular or bilabiate, with fleshy perianth segments. Inflorescences are racemes or panicles.	[24,25]
*Caprifoliaceae*	*Lonicera* *japonica* Thunb.	Japanese honeysuckle	Native to Asia; a twining, woody vine; the flowers, strongly two-lipped, initially white, turning yellow with a purple tint, and feature a hairy tube. It is blooming from May to July.	About 860 species, centered in eastern North America and East Asia, mostly of shrubs and vines. Flowers are tubular, funnel-shaped, or bell-like; inflorescences are thyrsoid, compact or loose, and often bracteate.	[26,27]
*Fabaceae*	*Clitoria ternatea* L.	butterfly pea, blue pea	Native to tropical Asia and Africa is a perennial herbaceous, growing as a vine or creeper, its flowers a vivid deep blue; solitary, with light yellow markings.	Over 19,000 species, flowers are monoecious, often in clusters, with a calyx of sepals and a colorful corolla; corolla with an upper banner petal and fused lower petals.	[28,29,30]
*Fabaceae*	*Robinia* *pseudoacacia* L.	black locust	A deciduous tree native to the eastern parts of North America; flowers are cream-white (rarely pink or purple) with a pale yellow blotch in the center and papilionaceous in shape. It blooms from May to June.	Please see above.	[31]
*Fabaceae*	*Trifolium pratense* L.	red clover, cow clover	A perennial herb native to Europe. Inflorescences are head-like with no peduncle. Flowers, generally red-purple, blooming from April to October.	Please see above.	[32]
*Iridaceae*	*Gladiolus grandiflorus* Andrews	sword-lily	A bulbous geophyte, native to the south-western and southern parts of South Africa; flowers, gathered in a spike, are cream to greenish with a darker stripe along the midrib, blooms from September to October.	With about 2000 species, nearly cosmopolitan; mostly perennial herbs; inflorescences in umbels, spikes, or solitary flowers. Flowers are pedicellate, with a pet-aloid perianth of two whorls of three sepals, often large.	[33,34]
*Lythreceae*	*Punica granatum* L.	pomegranate	Native to southeastern Europe, Asia, and the Himalayas, is a shrub or small tree; bright orange-red to pale yellow flowers, 2–3 cm across, bloom from April to July.	With about 600 species; mainly in the equatorial zone, extending to temperate regions; includes herbs, shrubs, or trees; flowers olitary or in inflorescences.	[35,36]
*Oleaceae*	*Forsythia* × *intermedia* Zabel	border forsythia	*Forsythia × intermedia* is a deciduous shrub; bright yellow, four-lobed flowers blooming in clusters in early spring, it thrives in full sun and various soils.	Over 600 species, is found in temperate and tropical zones; small flowers often have four or five fused petals, forming a tubular shape. Inflorescences vary, as cymes, panicles, racemes, umbels, or fascicles.	[37,38]
*Paeoniaceae*	*Paeonia* *lactiflora* Pall.	peony	A perennial herb, native to Asia including China (North-Central, South-Central, Southeast), Inner Mongolia, Manchuria, Mongolia, Siberia; characterized by white or pink flowers (wild varieties), of which there are usually several on the shoot.	Around 33 species in a single genus (Paeonia); native to the Northern Hemisphere; herbaceous perennials with large, flowers with radial symmetry in various colors have five or more layered petals.	[39,40]
*Plantaginaceae*	*Antirrhinum* *majus* L.	snapdragon	An annual or perennial plant native to the Mediterranean region; grows to 8–70 cm and terminal raceme inflorescences bear flowers of various colors; found in open, disturbed areas, it blooms from March to June.	Nearly 2000 species, bilaterally symmetrical flowers mostly found in temperate zones. The flowers small, with a tube-shaped corolla formed by five fused petals.	[41,42]
*Rubiaceae*	*Pentas* *lanceolata* Deflers	Egyptian starcluster	Native to Ethiopia to Mozambique, Comoros, Arabian Peninsula; a subshrub or shrub; five-petaled flowers may be red, white, lavender, purple, or shades of pink, sometimes two-toned.	Over 13,150 species, mainly found in tropical regions. Its flowers form inflorescences like cymes, heads, or panicles. The flowers are typically bisexual and radially symmetrical.	[43,44]

**Table 3:** Phytochemical constituents of chosen edible flowers.

Plant Species	Family	Compounds	References
*Antirrhinum majus*	*Plantaginaceae*	**Aurones**: bracteatin 6-glucoside **Carotenoids**: lutein, zeaxanthin, β-carotene **Chalcones**: chalcononaringenin 4′-glucoside, 3,4,2′,4′,6′-pentahydroxychalcone 4′-glucoside **Flavones**: apigenin 7,4′-diglucuronide, luteolin 7-glucuronide, chrysoeriol 7-glucuronide, kampferol 3-glucoside and kampferol 3,7-diglucoside **Flavonol glycosides**: quercetin-3-galactoside, quercetin-3-arabinofuranoside, quercetin-3-rhamnoside, quercetin-3-(6″-benzoyl)-β-galactoside, methoxyquercetin pentoside, and quercetin-3-(6″-coumaroyl)-β-galactoside **Terpenoids:** antirrhinoside, antirrhide, 5-glucosyl-antirrhinoside and linarioside (irydoids); myrcene, (E)-beta-ocimene (monorepenes) **Other compounds**: methyl benzoate	[49,55,56,70]
*Bellis perennis*	*Asteraceae*	**Anthocyanins**: glucuronylated and malonylated cyanidin-3-glucosides **Flavonoles**: kaempferol, quercetin, isorhamnetin, isorhamnetin-3-O-β-D-galactoside, isorhamnetin-3-O-β-D-(6″-acetyl)- galactoside, and kaempferol-3-O-β-D-glucoside **Flavones**: apigenin, apigenin-7-O-β-D-glucoside, apigenin-7-O-β-D-glucuronide, apigenin-7-O-(6″-E-caffeoyl)-β-D-glucoside, apigenin-7-O-β-Dmethylglucuronide **Phenolic acids**: caffeic, ferulic, sinapic, p-coumaric, and salicylic acids **Saponins**: triterpenoid saponins **Other compounds**: acetylenes, alcaloids, carbohydrates, proteins, steroids, tannins, terpenoids, volatiles (essential olil)	[71,108,109]
*Clitoria* *ternatea*	*Fabaceae*	**Anthocyanins**: delphinidin-3, 5-diglucoside, 3-O-β-glucoside,3-O-(2″-O-α-rahmnosyl)-β-glucoside, 3-O-(2″-O-α-rahmnosyl-6″-O-malonyl)-β-glucoside of delphinidin (delphinidin glycosides) **Fatty acids**: palmitic acid, stearic acids, petroselinic acids, linoleic acid, arachidic acid, behenic acid, phytanic acid **Flavonols**: quercetin, myricetin, and kaempferol derivatives (e.g., rutin) **Flavonol glycosides**: 3-O-(2″-O-α-rhamnosyl-6″-O-malonyl)-β-glucoside, 3-O-(6″-O-α-rhamnosyl-6″-O-malonyl)-β-glucoside, 3-O-(2″,6″-di-O-α-rhamnosyl)-β-glucoside of kaemferol, quercetin and myricetin **Phenolic acids**: ellagic acid **Phytosterols**: campesterol, stigmasterol, β-sitosterol, sitostanol **Terpenoids**: ternatin A1–A3, B1–B4, C1–C5, and D1–D3; preternatins A3 and C4; **Tocols**: α-tocopherol and -tocopherol	[59,63,110,111,112,113]
*Coreopsis* *tinctoria*	*Asteraceae*	**Chalcones:** butein, coreopsin, marein, okanin **Flavanones:** butin **Flavones**: flavanomarein, maritimein **Other coumpounds**: aurones, biflavonones, flavanoles, flavanonols, flavonols, flavonones, phenylpropanoids, polyacetylenes, polysaccharides	[73]
*Cosmos* *bipinnatus*	*Asteraceae*	**Aurones**: 7, 3′, 4′-trihydroxy-6-methoxyaurone **Chalcones**: 2′-hydroxy-4, 4′-dimethoxychalcone **Flavones glycosides**: chrysoeriol (5,7,4′-trihydroxy-3′-methoxyflavone)- 7-0-glucuronide, luteolin (5,7,3′, 4′-tetrahydroxyflavone)-7-0-glucuronide, 7, 2′, 5′-trihydroxyflavone	[114,115]
*Forsythia* × *intermedia*	*Oleaceae*	**Lignans**: isolariciresinol, phillygenin-O-glucoside (phyllyrin), arctigenin-Oglucoside (arctiin), matairesinol-O-glucoside (matairesinoside), pinoresinol-O-glucoside; (–) matairesinol-4′-o–D-glucoside (+) epipinoresinol-4′–D-glucoside (–)-secoisolariciresinol, (+)-pinoresinol-beta-D-glucoside, (+)-pinoresinol-beta-D-glucoside methyl ester **Lignan aglycones**: pinoresinol, epipinoresinol, matairesinol, phillygenin, arctigenin **Fatty acids**: stearic and palmitic acids **Flavone glycosides:** wogonin-7-O-glycoside **Flavonols glycosides**: rutin, kaempferol, rhamnohexoside, quercetin rhamnohexoside, quercetin-3-rhamnoglucoside, **Phenolic acids**: adoxosidic acid, chlorogenic acid, vanillic acid **Phenolic glycosides:** phillyrin, forsythoside, cornoside, **Phenylethanoids**: forsythoside A, verbascoside/acteoside **Phytosterols:** β-sitosterol hexoside, forsythoside A, **Other compounds:** sugar alcohols (erythritol), organic acids (succinic acid)	[75,116,117]
*Gladiolus grandiflorus*	*Iridaceae*	**Anthocyanins:** pelargonidin, cyanidin, peonidin, petunidin and malvidin glycosides cyanidin-3,5-diglucoside, delphinidin triglucoside delphinidin-3,5-diglucoside, malvidin-3-glucoside, malvidin-3-rhamnosylglucoside-5-glucoside, malvidin-3, 5-diglucoside, malvidin-3-rhamnosylglucoside-5-glucoside, malvidin-3-rhamnosylgucoside, pelargonidin 3-O-diglucoside-5-O–glucoside, pelargonidin-3-rhamnosylglucoside, pelargonidin-3-rhamnosylglucoside-5-glucoside, pelargonidin-3,5-diglucoside, pelargonidin-3-glucoside, peonidin-3,5-diglucoside, peonidin-3-rhamnosylglucoside, peonidin-3-rhamnosylglucoside- 5-glucoside, petunidin-3,5-diglucoside, petunidin-3-glucoside, petunidin-3-rhamnosylglucoside, petunidin-3-rhamnosylglucoside-5-glucoside, 3-O-rutinoside-5-O-glucosides of cyanidin, malvidin, pelargonidin and peonidin **Flavonol glycosides**: flavonol 3-O-glycosides of kaempferol, quercetin, myricetin, laricitrin and syringetin, kaempferol 3-O-rutinoside, kaempferol 3-O-sophoroside and quercetin 3-O-rutinoside (rutin) **Other compounds:** carotenoids	[64,118]
*Gomphrena globosa*	*Amaranthaceae*	**Betalains**: amaranthine, isoamaranthine, 17-decarboxy-amaranthine, gomphrenins and their derivatives, lampranthin II **Flavonols**: quercetin 3-O-(6-pentosyl)-hexoside, quercetin 3-O-rutinoside, kaempferol 3-O-(2-pentosyl, 6-O-rhamnosyl)- Hexoside, quercetin 3-O-glucoside, kaempferol 3-O-(2-pentosyl)-hexoside, kaempferol 3-O-rutinoside, isorhamnetin 3-O-rutinoside, kaempferol 3-O-glucoside, isorhamnetin 3-O-glucoside, quercetin O-glucuronide-O-hexoside, kaempferol O-acetylhexoside, gomphrenol 3-O-hexoside, gomphrenol 3-O-(6-acetyl)-hexoside, kaempferol 3-O-(2-rhamnosyl)-hexoside, kaempferol O-glucuronide-O-hexoside; chrysoeriol-7-O-beta-D-glucoside	[53,119,120,121,122,123,124,125,126]
		**Organic acids**: 9-Octadecenoic acid (Z)-, methyl ester, docosanoic acid, methyl ester, eicosanoic acid, methyl ester, hexadecanoic acid, methyl ester, methyl stearate, methyl tetradecanoate, octadecane, tetradecane, 3-ethyl-5-(2-ethylbutyl)-, phthalic acid, hept-4-yl isobutyl ester, methyl decanoate, methyl 6-hydroxy caproate hexanoic acid, nonanoic acid,7-methyl-methyl ester **Phenolic compounds**: cis-ferulic acid hexoside, trans-ferulic acid hexoside, cis-p-coumaric acid, trans-p-coumaric acid, cis-ferulic acid, trans-ferulic acid, gomphrenoside; gomphrenoside (phenols glycosides) **Sterols**: beta-sitosterol-3-O-beta-D-dlucoside, **Terpenoids**: hopane-7-bewta-ol, friedelin **Other compounds**: alcanes, alcohols, allantoin, alkaloids, aminoacids, anthocyanins, betalains, carbohydrates, coumarins, hydroxycinamamides, protein, saponins, steroids/triterpenoids, tannins; N, P, Ca, Mg, K, Fe, B, Zn, Cu, Mo, Mn;	
*Hemerocallis disticha*	*Asphodelaceae*	**Carotenoids**: neoxanthin, violaxanthin, violeoxanthin, 13-cis-lutein 5,6-epoxide, lutein 5,6-epoxide, 13-cis-lutein, all-trans-lutein, 9-cis-lutein, zeaxanthin, 13-cis-zeaxanthin, β-cryptoxanthin, 13-cis-β-cryptoxanthin, cis-β-cryptoxanthin, all-trans-β-carotene, 9-cis-β-carotene **Catechins**: catechin, epicatechin, (–)-epigallocatechin-3-gallate **Flavonols**: rutin, quercetin 3-arabinoside, kaempferol 3-glucoside, isorhamnetin 3-rutinoside, luteolin 7-glucoside, apigenin 7- glucoside **Isoflavones**: quercitrin **Phenolic acids**: caffeic acid, chlorogenic acid	[54,127,128,129,130]
*Lonicera* *japonica*	*Caprifoliaceae*	**Flavones**: chrysoeriol, chrysoeriol 7-O-D-glucopyranoside, luteolin, luteolin 7-O-D-glucoside; luteolin-7-O-D-galactoside, **Flavonols**: isorhamnetin 3-O-D-glucopyranoside; kaempferol 3-O-D-glucopyranoside; quercetin 3-O-D-glucopyranoside; **Phenolic acids**: caffeic acid, caffeoylquinic acids, oleanolic acid, protocatechuic acid **Saponins**: oleanane type and hederagenin type **Terpenoids:** linalool (monoterpene); (Z,Z)-farnesole (sesquiterpenoids), lonijaposide A1-A4 B1-B2, **Triterpenoid saponins**: 28-elfa-O-L-rhamnopyranosyl-(1→2)-[beta-D-xylopyranosyl(1→6)-beta-D-glucopyranosyol ester **Other compunds**: alcohols, aldehydes, alkaloids, amino acids, cerebrosides, ketones, micro and macroelements, nucleosides, hydrocarbons, 5-hydroxymethyl-2-furfural	[65,77,131,132]
*Paeonia* *lactiflora*	*Paeoniaceae*	**Anthocyanins:** pelargonidin 3-glucoside, cyanidin 3-glucoside, cyanidin 3,5-diglucoside, peonidin 3-glucoside, peonidin 3,5-Di-O-β-Dglucopyranoside, malvoside, **Flavonoids**: kaempferol-3-O-(6-O-galloyl)-β-Dglucopyranoside, kaempferol- 3-O-β-D-glucopyranosyl-7-O-α-Lrhamnopyranoside, quercetin-3-O-β-Dglucopyranoside, kaempferol-3, 7-di-O-β-D-glucopyranoside,quercetin-3-O-(6″-O-galloyl)-glucoside, astragalin, kaempferol-3-O-(6″-galloyl)-β-D-glucopyranosid, isorhamnetin-3-O-glucoside6″-gallate, astragalin, kaempferol 3,7-β-Ddiglucoside, populnin, lactifloraoside I, kaempferol 3,7-O-β-Ddiglucopyranosid, kaempherol 3-O-(2′′-galloyl)-β-Dglucopyranoside, kaempferol 3-O-(6′′-galloyl)-β-Dglucopyranoside, quercetin 3-O-(6′′ galloyl)-β-Dglucopyranoside, sexangulareinyle 3-O-β-D-sophoroside, limocitrinyle 3-O-β-Dsophoroside, isoquercitrin, isorhamnetin-3,7-diglucoside, kaempferol-3-O-β-D-glucoside, kaempferol-3,7-diglucoside, quercetin-3-O-D-(6″-Ogalloyl)-glucopyranoside **Phenolic acids:** benzoic acid, digallic acid, ellagic acid, gallic acid, 4-Hydroxybenzoic acid, isomer of salicylic acid, **Phenolic compounds**: phenethyl alcohol, methylarbutin, mudanpioside I, lactifloraoside I **Saponins**: astragaloside **Steroids**: β-sitosterol **Tannins**: 6-O-m-digalloyl-1,2,3,4-tetra-O-galloyl-β-D-glucopyranoside, 1,2,3,6-tetra-O-galloyl-β-D-glucopyranoside, methyl m-digallate and methyl p-digallate, ethyl m-digallate and ethyl p-digallate, 6-O-galloyl-Dglucopyranoside, 1-O-galloyl-β-Dglucopyranoside, 6′-O-galloylsucrose, 1′-Ogalloylsucrose, 1,2,3,4,6-penta-O-galloyl-β-D-glucopyranoside, 1-Galloyl-glucose, Glucogallin, 1,2,3,4,6-O-pentagalloylglucose, 1-O-Galloylglucose, 6-O-Galloylglucose, 1,3,6-tri-O-galloyl-β-D-glucose, 1,2,3,4,6-Pentagalloyl glucose, 1-O-Galloyl-β-D-glucose, 6,3′-Dimethoxyquercetindiglucoside, 1,2,3,6-Tetra-O-galloy-β-D–glucopyranose, hexagalloyl glucose **Terpenoids**: albiflorin, oxypaeoniflorin, paeoniflorin, floralalbiflorin I, benzoylpaeoniflorin, oxypaeoniflorin, ortho-oxypaeoniflorin, citronellal (rhodinal), citronellol **Other compunds**: ethyl gallate, methyl gallate, 2-phenylethyl-β-D-glucopyranoside, 2-phenylethyl)]-β-Dglucopyranosyl-(1→6)]-β-D-glucopyranoside, 2- phenylethyl-[α-L-rhamnopyranosyl-(1→6)]-β-Dglucopyranoside, 3-hydroxycitronellic acid-3-O-β-D-glucopyranoside, pentacosane, (Z)-Hex-3-en-1-ol, 13-Methyltetradecanoic acid, (Z)-Hex-3-enal, quinic acid, fatty acides derivatives, (Z)-(1S,5R)-β-pinene-10-yl-βcarotin, Ethyl-p-digallate, N, N′, N″-tris-(p-coumaroyl) spermidine	[67,79,133,134,135,136]
*Pentas* *lanceolata*	*Rubiaceae*	**Terpenoids**: asperuloside and asperulosidic acid tudoside, 13R-epi-gaertneroside, 13R-epiepoxygaertneroside, E-uenfoside and Z-uenfoside **Other compounds**: antraqiunones, saponin glucoside, alkaloids	[137,138]
*Punica* *granatum*	*Lythreceae*	**Anthocyanins**: pelargonidin 3,5-diglucoside and pelargonidin 3-glucoside **Fatty acids**: punicic acid **Flavonoids**: biochanin A, tricetin; punicaflavone **Phenolic acids**: ellagic acid, gallic acid **Sterols**: daucosterol, b-sitosterol **Tannins**: ethyl brevifolin-carboxylate, punicalagin, punicatannin C, **Triterpenes**: ursolic acid, oleanolic acid, maslinic acid, asiatic acid, **Other compounds**: punicanolic acid, galloyl glucoses, organic acids, brevifolin	[51,61,81,139,140,141,142,143]
*Robinia* *pseudoacacia*	*Fabaceae*	**Amino acids**: aspartic acid, threonine, serine, glutamic acid, glycine, alanine, cystine, valine, methionine, isoleucine, leucine, tyrosine, phenylalanine, lysine, histidine, arginine, proline, tryptophan Ascorbic acid, tannic acid **Fatty acids**: mostly palmitic, linoleic, linolenic, but also stearic, behenic, arachidic, oleic, heptadecanoic, docosahexaenoic, myristic, eicosapentaenoic, palmitic acid **Flavonoids**: 3-O-b-Glcp-(1→2)[a-Rhap-(1→6)]-b-Galp-7-O-a-Rhap, 3-O-b-Galp-7-O-a-Rhap, 3-O-a-Rhap-(1→6)-b-Galp-7-O-a-Rhap, 3-O-a-Rhap-(1→6)-b-Glcp-7-O-a-Rhap, 3-O-a-Rhap-(1→6)-b-Galp-7-O-a-Rhap, 3-O-a-Rhap-(1→6)-b-Galp, 7-O-a-Rhap, hyperoside and acacetin-7-O-ruthoside, kaempferol, acacetin, isophytol, quercetin, apigenin **Saponins**: triterpenoid glycosides—group A, B, E and DDMP saponins **Terpenes:** methyl benzene, 1-hexen-3-ol, dimethyl benzene, limonene, delra-carene, 2,6,10,14-tetramethylheptadecane, linalool ocide, dichlorobenzene, 2,5-dimethylfuran, 2-ethyl hexanol, linalool, alfa-terpineol, farnese, geraniol, benzyl alcohol, n-nonadecane, quinoline, n-heneicosane, docosane, methyl hexadecanoate, 2-methyl amino benzoate, ethyl undecanoate, 3,4-dimethoxy methyl benzoate, pentacosane, heptacosane, hexadecanoic acid, nonacosane, octadecanoic acid; trans-linalyl oxide; cis-linalyl oxide; methyl salicylate; p-anisic acid; n-pentadecane; n-hexadecane; methyl veratrate; cis-methyl dihydrojasmonate; n-heptadecane; (E,E)-farnesol; (E,Z)-farnesol; n-octadecane; isopropyl myristate; hexahydrofarnesyl acetone; diisobutyl phthalate; methyl isopalmitate; dibutyl phthalate; isopropyl hexadecanoate; tributyl acetylcitrate; n-pentacosane; diisooctyl phthalate; n-hexacosane; n-heptacosane; n-octacosane; all-trans-squalene; n-nonacosane; n-triacontane; n-hentriacontane; n-dotriacontane; β-amyrin; α-amyrin, monoterpenes as (Z)-ocimene and linalool, followed by geranyl nitrile and β-myrcene; α-pinene, β-pinene, β-myrcene, Z-ocimene, geranyl nitrile, and linalool. 2-butanone, 3-pentanone, and hexanal **Other compounds**: Ca, Cu, Fe, K, Mg, Na, P; free sugars: fructose, glucose, sucrose, fructose/glucose	[144,145,146,147,148,149]
*Trifolium pratense*	*Fabaceae*	**Coumarins**: (<0.03%) **Cyanogenic glucosides:** lotaustralin, linamarin **Flavonoids**: pterocarpans; flavon derivetives: naringenin **Isoflavonoids:** biochanin A, daidzein, genistein, formononetin, irilone, pratensein and prunetin, biochanin A-7-O-glucoside and formononetin-7-O-glucoside, biochanin A-7-O-glucoside-6″-O-malonate, daidzein-7-O-glucoside-6″-O-malonate, formononetin-7-O-glucoside-6″-O-malonate, genistein-7-O-glucoside-6″-O-malonate, irilone-40-O-glucoside-6″-O-malonate, pratensein-7-O-glucoside-6″-O-malonate and prunetin-40-O-glucoside-6″-O-malonate **Sterols**: coumestrol **Other compounds**: tyramine	[150]

## Data Availability

All data supporting reported results can be found within the manuscript and in the Authors.

## References

[1] Marrelli M., Statti G., Conforti F. (2020). A Review of Biologically Active Natural Products from Mediterranean Wild Edible Plants: Benefits in the Treatment of Obesity and Its Related Disorders. Molecules.

[2] European Medicines Agency (2017). Final European Union Herbal Monograph on Calendula Officinalis L., Flos (Revision 1). EMA/HMPC/98717/2014. https://www.Ema.Europa.Eu/En/Medicines/Herbal/Calendulae-Flos.

[3] European Medicines Agency (2008). Final Assessment Report on Matricaria Recutita L., Flos and Matricaria Recutita L., Aetheroleum (First Version). EMA/HMPC/196814/2006. https://www.ema.europa.eu/en/medicines/herbal/matricariae-flos.

[4] European Medicines Agency (2014). Final European Union Herbal Monograph on Sambucus Nigra L., Flos (Revision 1). EMA/HMPC/442085/2012. https://www.ema.europa.eu/en/medicines/herbal/sambuci-flos.

[5] European Medicines Agency (2016). Final European Union Herbal Monograph on Verbascum Thapsus L., V. Densiflorum Bertol., V. Thapsiforme Schrad. and V. Phlomoides L., Flos. EMA/HMPC/43778/2015. https://www.ema.europa.eu/en/medicines/herbal/verbasci-flos.

[6] European Medicines Agency (2012). Final Assessment Report on Lavandula Angustifolia Miller, Aetheroleum and Lavandula Angustifolia Miller, Flos. EMA/HMPC/127843/2011. https://www.ema.europa.eu/en/medicines/herbal/lavandulae-flos.

[7] Mn D., Tr S., Vendrapati R., Reddy M.P., Gaddam V. (2015). A Review on the Therapeutic Uses of Flowers as Depicted in Classical Texts of Ayurveda and Siddha. J. Res. Educ. Indian Med..

[8] Santos I.C.D., Reis S.N. (2021). Edible Flowers: Traditional and Current Use. Ornam. Hortic..

[9] Mlcek J., Rop O. (2011). Fresh Edible Flowers of Ornamental Plants—A New Source of Nutraceutical Foods. Trends Food Sci. Technol..

[10] Skrajda-Brdak M., Dąbrowski G., Konopka I. (2020). Edible Flowers, a Source of Valuable Phytonutrients and Their pro-Healthy Effects—A Review. Trends Food Sci. Technol..

[11] Fernandes L., Casal S., Pereira J.A., Saraiva J.A., Ramalhosa E. (2017). Edible Flowers: A Review of the Nutritional, Antioxidant, Antimicrobial Properties and Effects on Human Health. J. Food Compos. Anal..

[12] Chensom S., Okumura H., Mishima T. (2019). Primary Screening of Antioxidant Activity, Total Polyphenol Content, Carotenoid Content, and Nutritional Composition of 13 Edible Flowers from Japan. Prev. Nutr. Food Sci..

[13] Topolska K., Florkiewicz A., Filipiak-Florkiewicz A. (2021). Functional Food—Consumer Motivations and Expectations. Int. J. Environ. Res. Public Health.

[14] Pires T.C.S.P., Barros L., Santos-Buelga C., Ferreira I.C.F.R. (2019). Edible Flowers: Emerging Components in the Diet. Trends Food Sci. Technol..

[15] Kumari P., Kashyap U., Bhargava B. (2021). Phytochemicals from Edible Flowers: Opening a New Arena for Healthy Lifestyle. J. Funct. Foods.

[16] Keil D.J. Bellis Perennis, in Jepson Flora Project (Eds.) Jepson eFlora. https://ucjeps.berkeley.edu/eflora/eflora_display.php?tid=1652.

[17] WFO Asteraceae Giseke. http://www.worldfloraonline.org/taxon/wfo-7000000146.

[18] Keil D.J. Coreopsis Tinctoria, in Jepson Flora Project (Eds.) Jepson eFlora. https://ucjeps.berkeley.edu/eflora/eflora_display.php?tid=2365.

[19] Keil D.J. Cosmos Bipinnatus, in Jepson Flora Project (Eds.) Jepson eFlora. https://ucjeps.berkeley.edu/eflora/eflora_display.php?tid=2385.

[20] Missouri Botanical Garden Gomphrena Globosa. https://www.missouribotanicalgarden.org/PlantFinder/PlantFinderDetails.aspx?kempercode=a115&utm_source=chatgpt.com.

[21] Cai Y.-Z., Xing J., Sun M., Corke H. (2006). Rapid Identification of Betacyanins from *Amaranthus tricolor*, *Gomphrena globosa*, and *Hylocereus polyrhizus* by Matrix-Assisted Laser Desorption/Ionization Quadrupole Ion Trap Time-of-Flight Mass Spectrometry (MALDI-QIT-TOF MS). J. Agric. Food Chem..

[22] Silva L.R., Valentão P., Faria J., Ferreres F., Sousa C., Gil-Izquierdo A., Pinho B.R., Andrade P.B. (2012). Phytochemical Investigations and Biological Potential Screening with Cellular and Non-Cellular Models of Globe Amaranth (*Gomphrena globosa* L.) Inflorescences. Food Chem..

[23] Townsend C.C., Kubitzki K., Rohwer J.G., Bittrich V. (1993). Amaranthaceae. Flowering Plants · Dicotyledons.

[24] Royal Botanic Gardens Kew, Plants of the World Online Hemerocallis Fulva Var. Angustifolia Baker. https://powo.science.kew.org/taxon/urn:lsid:ipni.org:names:77189035-1#other-data.

[25] Smith G.F., Van Wyk B.-E., Kubitzki K. (1998). Asphodelaceae. Flowering Plants · Monocotyledons.

[26] Bell C.D., Lauramay T.D. Lonicera Japonica, in Jepson Flora Project (Eds.) Jepson eFlora. https://ucjeps.berkeley.edu/eflora/eflora_display.php?tid=31508.

[27] WFO Caprifoliaceae Juss. http://www.worldfloraonline.org/taxon/wfo-7000000112.

[28] Oguis G.K., Gilding E.K., Jackson M.A., Craik D.J. (2019). Butterfly Pea (Clitoria Ternatea), a Cyclotide-Bearing Plant with Applications in Agriculture and Medicine. Front. Plant Sci..

[29] Missouri Botanical Garden Clitoria Ternatea. https://www.missouribotanicalgarden.org/PlantFinder/PlantFinderDetails.aspx?taxonid=280445.

[30] WFO Fabaceae Juss. http://www.worldfloraonline.org/taxon/wfo-7000000323.

[31] Lavin M., Isely D., McClinrock E. Robinia Pseudoacacia, in Jepson Flora Project (Eds.) Jepson eFlora. https://ucjeps.berkeley.edu/eflora/eflora_display.php?tid=41521.

[32] Vincent M.A. Trifolium Pratense, in Jepson Flora Project (Eds.) Jepson eFlora, Revision 12. https://ucjeps.berkeley.edu/eflora/eflora_display.php?tid=47160.

[33] Royal Botanic Gardens Kew, Plants of the World Online Gladiolus Grandiflorus Andrews. https://powo.science.kew.org/taxon/urn:lsid:ipni.org:names:437495-1.

[34] Venditti A., Guarcini L., Ballero M., Bianco A. (2015). Iridoid Glucosides from Pentas Lanceolata (Forssk.) Deflers Growing on the Island of Sardinia. Plant Syst. Evol..

[35] Graham S.A. Punica Granatum, in Jepson Flora Project (Eds.) Jepson eFlora. https://ucjeps.berkeley.edu/eflora/eflora_display.php?tid=40368.

[36] WFO Lythraceae J.St.-Hil. http://www.worldfloraonline.org/taxon/wfo-7000000356.

[37] Brand M.H. University of Connecticut Plant Database Forsythia x Intermedia. https://plantdatabase.uconn.edu/detail.php?pid=176.

[38] WFO Oleaceae Hoffmanns. & Link. http://www.worldfloraonline.org/taxon/wfo-7000000422.

[39] WFO Paeonia Lactiflora Pall. http://www.worldfloraonline.org/taxon/wfo-0000480479.

[40] WFO Paeoniaceae Raf. http://www.worldfloraonline.org/taxon/wfo-7000000436.

[41] Keil D.J., Wetherwax M., Thompson D.M. Antirrhinum Majus in Jepson Flora Project (Eds.) Jepson eFlora, Revision 8. https://ucjeps.berkeley.edu/eflora/eflora_display.php?tid=13557.

[42] WFO Plantaginaceae Juss. http://www.worldfloraonline.org/taxon/wfo-7000000476.

[43] Royal Botanic Gardens Kew, Plants of the World Online Pentas Lanceolata (Forssk.) Deflers. https://powo.science.kew.org/taxon/urn:lsid:ipni.org:names:760275-1#children.

[44] WFO Rubiaceae Juss. http://www.worldfloraonline.org/taxon/wfo-7000000534.

[45] Pensamiento-Niño C.A., Castañeda-Ovando A., Añorve-Morga J., Hernández-Fuentes A.D., Aguilar-Arteaga K., Ojeda-Ramírez D. (2024). Edible Flowers and Their Relationship with Human Health: Biological Activities. Food Rev. Int..

[46] Wang Y. (2013). In Vitro Antioxidant Capacity of Daylily (*Hemerocallis disticha*) Flowers Cultivated in Taiwan. Life Sci. J..

[47] Nelsen J., Ulbricht C., Barrette E.P., Mac D.S., Tsouronis C., Rogers A., Basch S., Hashmi S., Bent S., Basch E. (2002). Red Clover (Trifolium Pratense) Monograph: A Clinical Decision Support Tool. J. Herbal. Pharmacother..

[48] Tarnam Y.A., Ilyas M.H.M., Begum T.N. (2014). Biological Potential and Phytopharmacological Screening of Gomphrena Species. Int. J. Pharma Res. Rev..

[49] Al-Snafi A. (2015). Therapeutic Properties of Medicinal Plants: A Review of Medicinal Plants with Central Nervous Effects (Part 1). Int. J. Pharmacol. Toxicol..

[50] Cakilcioglu U., Khatun S., Turkoglu I., Hayta S. (2011). Ethnopharmacological Survey of Medicinal Plants in Maden (Elazig-Turkey). J. Ethnopharmacol..

[51] Ahmad I., Zahin M., Aqil F., Hasan S., Khan M.S.A., Owais M. (2008). Bioactive Compounds from Punica Granatum, Curcuma Longa and Zingiber Officinale and Their Therapeutic Potential. Drugs Future.

[52] Malca-Garcia G.R., Zagal D., Graham J., Nikolić D., Friesen J.B., Lankin D.C., Chen S.-N., Pauli G.F. (2019). Dynamics of the Isoflavone Metabolome of Traditional Preparations of *Trifolium pratense* L.. J. Ethnopharmacol..

[53] Sherif A.T.Y. (2021). In Vitro Evaluation of Purple Inflorescence of Gomphrena Globosa (L.) Extracts for Antiinflammatory Activity and Its GC/MS Profile. AJBLS.

[54] Lu B., Li M., Yin R. (2016). Phytochemical Content, Health Benefits, and Toxicology of Common Edible Flowers: A Review (2000–2015). Crit. Rev. Food Sci. Nutr..

[55] Seo J., Lee J., Yang H.Y., Ju J. (2020). *Antirrhinum majus* L. Flower Extract Inhibits Cell Growth and Metastatic Properties in Human Colon and Lung Cancer Cell Lines. Food Sci. Nutr..

[56] Al-Snafi A. (2015). The Pharmacological Importance of Antirrhinum Majus—A Review. Asian J. Pharm. Sci. Tech..

[57] Oliveira H.B.D., Kffuri C.W., Casali V.W.D. (2010). Ethnopharmacological Study of Medicinal Plants Used in Rosário Da Limeira, Minas Gerais, Brazil. Rev. Bras. Farm..

[58] Vogl S., Picker P., Mihaly-Bison J., Fakhrudin N., Atanasov A.G., Heiss E.H., Wawrosch C., Reznicek G., Dirsch V.M., Saukel J. (2013). Ethnopharmacological in Vitro Studies on Austria’s Folk Medicine—An Unexplored Lore in Vitro Anti-Inflammatory Activities of 71 Austrian Traditional Herbal Drugs. J. Ethnopharmacol..

[59] Mukherjee P.K., Kumar V., Kumar N.S., Heinrich M. (2008). The Ayurvedic Medicine Clitoria Ternatea—From Traditional Use to Scientific Assessment. J. Ethnopharmacol..

[60] Hsu H.-F., Hsiao P.-C., Kuo T.-C., Chiang S.-T., Chen S.-L., Chiou S.-J., Ling X.-H., Liang M.-T., Cheng W.-Y., Houng J.-Y. (2016). Antioxidant and Anti-Inflammatory Activities of Lonicera Japonica Thunb. Var. Sempervillosa Hayata Flower Bud Extracts Prepared by Water, Ethanol and Supercritical Fluid Extraction Techniques. Ind. Crops Prod..

[61] Bekir J., Mars M., Vicendo P., Fterrich A., Bouajila J. (2013). Chemical Composition and Antioxidant, Anti-Inflammatory, and Antiproliferation Activities of Pomegranate (*Punica granatum*) Flowers. J. Med. Food.

[62] Gupta G., Chahal J., Bhatia M. (2010). Clitoria Ternatea (L.): Old and New Aspects. J. Pharm. Res..

[63] Jeyaraj E.J., Lim Y.Y., Choo W.S. (2021). Extraction Methods of Butterfly Pea (Clitoria Ternatea) Flower and Biological Activities of Its Phytochemicals. J. Food Sci. Technol..

[64] Lim T.K. (2014). Gladiolus Grandiflorus. Edible Medicinal and Non Medicinal Plants.

[65] Shang X., Pan H., Li M., Miao X., Ding H. (2011). Lonicera Japonica Thunb.: Ethnopharmacology, Phytochemistry and Pharmacology of an Important Traditional Chinese Medicine. J. Ethnopharmacol..

[66] Wang T., Xie A., Zhang D., Liu Z., Li X., Li Y., Sun X. (2021). Analysis of the Volatile Components in Flowers of Paeonialactiflora Pall. and Paeonialactiflora Pall. var. Trichocarpa. AJPS.

[67] Zhou H., Li T., Li B., Sun S. (2023). Skin Health Properties of Paeonia Lactiflora Flower Extracts and Tyrosinase Inhibitors and Free Radical Scavengers Identified by HPLC Post-Column Bioactivity Assays. Heliyon.

[68] Fu M., He Z., Zhao Y., Yang J., Mao L. (2009). Antioxidant Properties and Involved Compounds of Daylily Flowers in Relation to Maturity. Food Chem..

[69] Lucchetti L., Zitti S., Taffetani F. (2019). Ethnobotanical Uses in the Ancona District (Marche Region, Central Italy). J. Ethnobiol. Ethnomed..

[70] González-Barrio R., Periago M.J., Luna-Recio C., Garcia-Alonso F.J., Navarro-González I. (2018). Chemical Composition of the Edible Flowers, Pansy (Viola Wittrockiana) and Snapdragon (Antirrhinum Majus) as New Sources of Bioactive Compounds. Food Chem..

[71] Karakas F.P., Turker A.U., Karakas A., Mshvildadze V., Pichette A., Legault J. (2017). In Vitro Cytotoxic, Antibacterial, Anti-Inflammatory and Antioxidant Activities and Phenolic Content in Wild-Grown Flowers of Common Daisy—A Medicinal Plant. J. Herbal. Med..

[72] Afrianto W.F., Tamnge F., Hasanah L.N. (2020). Review: A Relation between Ethnobotany and Bioprospecting of Edible Flower Butterfly Pea (Clitoria Ternatea) in Indonesia. Asian J. Ethnobiol..

[73] Shen J., Hu M., Tan W., Ding J., Jiang B., Xu L., Hamulati H., He C., Sun Y., Xiao P. (2021). Traditional Uses, Phytochemistry, Pharmacology, and Toxicology of Coreopsis Tinctoria Nutt.: A Review. J. Ethnopharmacol..

[74] Saleem M., Ali H.A., Akhtar M.F., Saleem U., Saleem A., Irshad I. (2019). Chemical Characterisation and Hepatoprotective Potential of *Cosmos sulphureus* Cav. and *Cosmos bipinnatus* Cav. Nat. Product. Res..

[75] Michalak B., Filipek A., Chomicki P., Pyza M., Woźniak M., Żyżyńska-Granica B., Piwowarski J.P., Kicel A., Olszewska M.A., Kiss A.K. (2018). Lignans from Forsythia x Intermedia Leaves and Flowers Attenuate the Pro-Inflammatory Function of Leukocytes and Their Interaction with Endothelial Cells. Front. Pharmacol..

[76] Arthi V., Prasanna G. (2016). Hptlc Finger Print Profile and in Vitro Antioxidant Activity of Gomphrena Globosa L. Flowers. Int. J. Pharm. Sci. Rev. Res..

[77] Peng L.-Y., Mei S.-X., Jiang B., Zhou H., Sun H.-D. (2000). Constituents from Lonicera Japonica. Fitoterapia.

[78] Lim T.K. (2014). Paeonia Lactiflora. Edible Medicinal and Non Medicinal Plants.

[79] Ogawa K., Nakamura S., Sugimoto S., Tsukioka J., Hinomaru F., Nakashima S., Matsumoto T., Ohta T., Fujimoto K., Yoshikawa M. (2015). Constituents of Flowers of Paeoniaceae Plants, Paeonia Suffruticosa and Paeonia Lactiflora. Phytochem. Lett..

[80] Shaygannia E., Bahmani M., Zamanzad B., Rafieian-Kopaei M. (2016). A Review Study on *Punica granatum* L.. J. Evid. Based Complement. Altern. Med..

[81] Maphetu N., Unuofin J.O., Masuku N.P., Olisah C., Lebelo S.L. (2022). Medicinal Uses, Pharmacological Activities, Phytochemistry, and the Molecular Mechanisms of Punica Granatum L. (Pomegranate) Plant Extracts: A Review. Biomed. Pharmacother..

[82] Vitasović-Kosić I., Kaligarič M., Juračak J. (2021). Divergence of Ethnobotanical Knowledge of Slovenians on the Edge of the Mediterranean as a Result of Historical, Geographical and Cultural Drivers. Plants.

[83] Semwal R.B., Semwal D.K., Combrinck S., Viljoen A.M. (2015). Gingerols and Shogaols: Important Nutraceutical Principles from Ginger. Phytochemistry.

[84] Zhang H., Tsao R. (2016). Dietary Polyphenols, Oxidative Stress and Antioxidant and Anti-Inflammatory Effects. Curr. Opin. Food Sci..

[85] Milani A., Basirnejad M., Shahbazi S., Bolhassani A. (2017). Carotenoids: Biochemistry, Pharmacology and Treatment. Br. J. Pharmacol..

[86] Stahl W., Sies H. (2003). Antioxidant Activity of Carotenoids. Mol. Asp. Med..

[87] Cox-Georgian D., Ramadoss N., Dona C., Basu C., Joshee N., Dhekney S.A., Parajuli P. (2019). Therapeutic and Medicinal Uses of Terpenes. Medicinal Plants.

[88] Nouri A., Mofasseri M., Jahani R., Ghodrati M., Emam S.M.M., Ebadi M. (2024). Phytochemical Composition, Hypnotic Activity, and Antinociceptive Properties of Cumin Essential Oil Collected from Various Geographical Regions. Food Sci. Nutr..

[89] Bhavaniramya S., Vishnupriya S., Al-Aboody M.S., Vijayakumar R., Baskaran D. (2019). Role of Essential Oils in Food Safety: Antimicrobial and Antioxidant Applications. Grain Oil Sci. Technol..

[90] Sharifi-Rad J., Sureda A., Tenore G., Daglia M., Sharifi-Rad M., Valussi M., Tundis R., Sharifi-Rad M., Loizzo M., Ademiluyi A. (2017). Biological Activities of Essential Oils: From Plant Chemoecology to Traditional Healing Systems. Molecules.

[91] Li C., Zhu H., Zhao K., Li X., Tan Z., Zhang W., Cai Q., Wu X., Mo J., Zhang L. (2022). Chemical Constituents, Biological Activities and Anti-rheumatoid Arthritic Properties of Four *Citrus* Essential Oils. Phytother. Res..

[92] Kowalczyk A., Przychodna M., Sopata S., Bodalska A., Fecka I. (2020). Thymol and Thyme Essential Oil—New Insights into Selected Therapeutic Applications. Molecules.

[93] Tomi K., Kitao M., Murakami H., Matsumura Y., Hayashi T. (2018). Classification of Lavender Essential Oils: Sedative Effects of *Lavandula* Oils. J. Essent. Oil Res..

[94] Heghes S.C., Vostinaru O., Rus L.M., Mogosan C., Iuga C.A., Filip L. (2019). Antispasmodic Effect of Essential Oils and Their Constituents: A Review. Molecules.

[95] Gakuubi M., Wanzala W., Wagacha M., Dossaji S. (2016). Bioactive Properties of Tagetes Minuta L. (Asteraceae) Essential Oils: A Review. Am. J. Essent. Oils Nat. Prod..

[96] Stanic G., Samaržija I. (1993). Diuretic Activity of *Satureja Montana* Subsp. Montana Extracts and Oil in Rats. Phytother. Res..

[97] Galas A., Niemiec R., Matysek M., Olszanicka A., Kołodziejczyk K., Hop I., Branewska J., Ostrowska B., Imioło J., Maciąg A. (2023). Multidirectional Pharmacological Effects of Saponins in Light of Clinical Trials: A Systematic Review of Literature. J. Educ. Health Sport..

[98] Takahashi J.A., Rezende F.A.G.G., Moura M.A.F., Dominguete L.C.B., Sande D. (2020). Edible Flowers: Bioactive Profile and Its Potential to Be Used in Food Development. Food Res. Int..

[99] Güçlü-Üstündağ Ö., Mazza G. (2007). Saponins: Properties, Applications and Processing. Crit. Rev. Food Sci. Nutr..

[100] Abed El Aziz M. (2019). A Review on Saponins from Medicinal Plants: Chemistry, Isolation, and Determination. J. Nanomed. Res..

[101] Topka P., Rudzińska M., Poliński S., Szydłowska-Czerniak A., Tańska M. (2024). Enhancing Antioxidant Activity and Nutritional Profile of Dark Chocolate Through Enrichment with Plant Sterols: A Study on Phytosterol Concentrations and Functional Properties. Foods.

[102] Zio S., Tarnagda B., Tapsoba F., Zongo C., Savadogo A. (2024). Health Interest of Cholesterol and Phytosterols and Their Contribution to One Health Approach: Review. Heliyon.

[103] Rustan A.C., Drevon C.A. (2005). Fatty Acids: Structures and Properties. Encyclopedia of Life Sciences.

[104] Adamczak A., Ożarowski M., Karpiński T.M. (2019). Antibacterial Activity of Some Flavonoids and Organic Acids Widely Distributed in Plants. J. Clin. Med..

[105] Kumar V., Sharma A., Kaur R., Thukral A.K., Bhardwaj R., Ahmad P. (2017). Differential Distribution of Amino Acids in Plants. Amino Acids.

[106] Halder M., Jha S. (2023). Medicinal Plants and Bioactive Phytochemical Diversity: A Fountainhead of Potential Drugs Against Human Diseases. Medicinal Plants: Biodiversity, Biotechnology and Conservation.

[107] Benvenuti S., Mazzoncini M. (2021). The Biodiversity of Edible Flowers: Discovering New Tastes and New Health Benefits. Front. Plant Sci..

[108] Siatka T., Kašparová M. (2010). Seasonal Variation in Total Phenolic and Flavonoid Contents and DPPH Scavenging Activity of Bellis Perennis L. Flowers. Molecules.

[109] Avato P., Tava A. (1995). Acetylenes and Terpenoids of Bellis Perennis. Phytochemistry.

[110] Lijon M. (2017). Phytochemistry and Pharmacological Activities of Clitoria Ternatea. Int. J. Nat. Soc. Sci..

[111] Al-Snafi A. (2016). Pharmacological Importance of Clitoria Ternatea—A Review. IOSR J. Pharm..

[112] Jamil N., Zairi M.N.M., Nasim N.A.I.M., Pa’ee F. (2018). Influences of Environmental Conditions to Phytoconstituents in Clitoria Ternatea (Butterfly Pea Flower)—A Review. JST.

[113] Jeyaraj E.J., Lim Y.Y., Choo W.S. (2022). Antioxidant, Cytotoxic, and Antibacterial Activities of Clitoria Ternatea Flower Extracts and Anthocyanin-Rich Fraction. Sci. Rep..

[114] Hoang M.H., Vo T.N. (2016). Flavonoids and Chalconoid Isolated from Flowers of Cosmos Bipinnatus Cav. (Asteraceae). J. Tech. Educ. Sci..

[115] Saito K. (1976). Flavone Glycosides in the Ray Flowers of *Cosmos bipinnatus*. Planta Med..

[116] Zhang Q., Jia C., Xu H., Wang Y., Zhang M., Huo C., Shi Q., Yu S. (2012). Chemical Constituents of Plants from the Genus Forsythia. Mini-Rev. Org. Chem..

[117] Gavrilin M.V., Senchenko S.P. (2014). Identification of a Number of Flavolignans and Assay of Forsythoside a in the Flowers of Forsythia Intermedia (Forsythia X Intermedia Zabel) by HPLC/MS and Capillary Electrophoresis. Pharm. Chem. J..

[118] Souza A.G.D., Jung E.A., Benedicto V.P., Bosco L.C. (2021). Bioactive Compounds in Gladiolus Flowers. Ornam. Hortic..

[119] Spórna-Kucab A., Hołda E., Wybraniec S. (2016). High-Speed Counter-Current Chromatography in Separation of Betacyanins from Flowers of Red Gomphrena Globosa L. Cultivars. J. Chromatogr. B.

[120] Dinda B., Ghosh B., Achari B., Arima S., Sato N., Harigaya Y. (2006). Chemical Constituents of Gomphrena Globosa. II. Nat. Prod. Sci..

[121] Roriz C.L., Barros L., Carvalho A.M., Santos-Buelga C., Ferreira I.C.F.R. (2014). Pterospartum Tridentatum, Gomphrena Globosa and Cymbopogon Citratus: A Phytochemical Study Focused on Antioxidant Compounds. Food Res. Int..

[122] Dias D., Arcanjo D., Albuquerque C., Neto B., Lopes L., Santana R., Castelo N., Silva B., Moita M., Das M. (2011). Phytochemical Screening and Evaluation of Cytotoxic, Antimicrobial and Cardiovascular Effects of Gomphrena Globosa L. (Amaranthaceae). J. Med. Plant Res..

[123] Drobnicka N., Sutor K., Kumorkiewicz-Jamro A., Spórna-Kucab A., Antonik M., Dziedzic E., Świergosz T., Ortyl J., Wybraniec S. (2020). Phytochemical Molecules from the Decarboxylation of Gomphrenins in Violet Gomphrena Globosa L.—Floral Infusions from Functional Food. Int. J. Mol. Sci..

[124] Roriz C.L., Barros L., Prieto M.A., Morales P., Ferreira I.C.F.R. (2017). Floral Parts of Gomphrena Globosa L. as a Novel Alternative Source of Betacyanins: Optimization of the Extraction Using Response Surface Methodology. Food Chem..

[125] Heuer S., Wray V., Metzger J.W., Strack D. (1992). Betacyanins from Flowers of Gomphrena Globosa. Phytochemistry.

[126] Dhalaria R., Verma R., Sharma R., Jomova K., Nepovimova E., Kumar H., Kuca K. (2024). Assessing the Potential Role of Arbuscular Mycorrhizal Fungi in Improving the Phytochemical Content and Antioxidant Properties in Gomphrena Globosa. Sci. Rep..

[127] Hsu Y.-W., Tsai C.-F., Chen W.-K., Ho Y.-C., Lu F.-J. (2011). Determination of Lutein and Zeaxanthin and Antioxidant Capacity of Supercritical Carbon Dioxide Extract from Daylily (Hemerocallis Disticha). Food Chem..

[128] Wang Y., Xu T., Fan B., Zhang L., Lu C., Wang D., Liu X., Wang F. (2018). Advances in Researches on Chemical Composition and Functions of Hemerocallis Plants. Med. Plant.

[129] Li S., Cui H., Wang J., Hou F., Xiong X., Kang X., Xing G. (2021). Qualitative and Quantitative Analysis on Flavonoid Distribution in Different Floral Parts of 42 Hemerocallis Accessions. Front. Plant Sci..

[130] Tai C.-Y., Chen B.H. (2000). Analysis and Stability of Carotenoids in the Flowers of Daylily (*Hemerocallis d Isticha*) as Affected by Various Treatments. J. Agric. Food Chem..

[131] Tang X., Liu X., Zhong J., Fang R. (2021). Potential Application of Lonicera Japonica Extracts in Animal Production: From the Perspective of Intestinal Health. Front. Microbiol..

[132] Lim T.K. (2014). Lonicera Japonica. Edible Medicinal and Non-Medicinal Plants.

[133] Li P., Shen J., Wang Z., Liu S., Liu Q., Li Y., He C., Xiao P. (2021). Genus Paeonia: A Comprehensive Review on Traditional Uses, Phytochemistry, Pharmacological Activities, Clinical Application, and Toxicology. J. Ethnopharmacol..

[134] Wu Y., Jiang Y., Zhang L., Zhou J., Yu Y., Zhou Y., Kang T. (2021). Chemical Profiling and Antioxidant Evaluation of *Paeonia lactiflora* Pall. “Zhongjiang” by HPLC–ESI–MS Combined with DPPH Assay. J. Chromatogr. Sci..

[135] Magid A.A., Schmitt M., Prin P.-C., Pasquier L., Voutquenne-Nazabadioko L. (2017). In Vitro Tyrosinase Inhibitory and Antioxidant Activities of Extracts and Constituents of Paeonia Lactiflora Pall. Flowers. NPJ.

[136] Zhao Q., Gu L., Li Y., Zhi H., Luo J., Zhang Y. (2023). Volatile Composition and Classification of Paeonia Lactiflora Flower Aroma Types and Identification of the Fragrance-Related Genes. Int. J. Mol. Sci..

[137] Abd-Alla H.I., Sweelam H.M., Mohamed T.A., Gabr M.M., El-Safty M.M., Hegazy M.-E.F. (2017). Efficacy of Extracts and Iridoid Glucosides from Pentas Lanceolata on Humoral and Cell-Mediated Immune Response of Viral Vaccine. Med. Chem. Res..

[138] Schripsema J., Caprini C.P., Heijden R., Bino R., De Vos R., Dagnino D. (2007). Iridoids from Pentas Lanceolata. J. Nat. Prod..

[139] Ge S., Duo L., Wang J., GegenZhula, Yang J., Li Z., Tu Y. (2021). A Unique Understanding of Traditional Medicine of Pomegranate, Punica Granatum L. and Its Current Research Status. J. Ethnopharmacol..

[140] Arun N., Singh D.P. (2012). Punica Granatum: A Review on Pharmacological and Therapeutic Properties. J. Pharm. Sci. Res..

[141] Zehra T., Ahmed S.A., Zehra S.A. (2019). Review of Characteristic Components, Traditional and Pharmacological Properties of Punica Granatum. RADS J. Pharm. Pharm. Sci..

[142] Gościniak A., Bazan-Woźniak A., Pietrzak R., Cielecka-Piontek J. (2022). Pomegranate Flower Extract—The Health-Promoting Properties Optimized by Application of the Box–Behnken Design. Molecules.

[143] Gościniak A., Rosiak N., Szymanowska D., Miklaszewski A., Cielecka-Piontek J. (2024). Prebiotic Systems Containing Anthocyanin-Rich Pomegranate Flower Extracts with Antioxidant and Antidiabetic Effects. Pharmaceutics.

[144] Kwon J.-H., Byun M.-W., Kim Y.-H. (1995). Chemical Composition of Acacia Flower(Robinia Pseudo-Acacia). Korean J. Food Sci. Technol..

[145] Ma X., Chen J., Lu X., Zhe Y., Jiang Z. (2021). HPLC Coupled with Quadrupole Time of Flight Tandem Mass Spectrometry for Analysis of Glycosylated Components from the Fresh Flowers of Two Congeneric Species: *Robinia hispida* L. and *Robinia pseudoacacia* L.. J. Sep. Sci..

[146] Veitch N.C., Elliott P.C., Kite G.C., Lewis G.P. (2010). Flavonoid Glycosides of the Black Locust Tree, Robinia Pseudoacacia (Leguminosae). Phytochemistry.

[147] C Lina D., Olah N.K., P Tru E., Docea A., Popescu H., Bubulica M.-V. (2013). Chromatographic Analysis of the Flavonoids from Robinia Pseudoacacia Species. Curr. Health Sci. J..

[148] Aronne G., Giovanetti M., Sacchi R., De Micco V. (2014). From Flower to Honey Bouquet: Possible Markers for the Botanical Origin of *Robinia* Honey. Sci. World J..

[149] Stankov S., Fidan H., Ivanova T., Stoyanova A., Damyanova S., Desyk M. (2018). Chemical Composition and Application of Flowers of False Acacia (Robinia Pseudoacacia L.). Ukr. Food J..

[150] Kolodziejczyk-Czepas J. (2016). *Trifolium* Species—The Latest Findings on Chemical Profile, Ethnomedicinal Use and Pharmacological Properties. J. Pharm. Pharmacol..

[151] Prabawati N.B., Oktavirina V., Palma M., Setyaningsih W. (2021). Edible Flowers: Antioxidant Compounds and Their Functional Properties. Horticulturae.

[152] Sood Y., Lal M., Kalia A., Verma S. (2024). Edible Flowers: Super Foods with Potential Health Benefits. IJPSS.

[153] Pisoschi A.M., Pop A., Cimpeanu C., Predoi G. (2016). Antioxidant Capacity Determination in Plants and Plant-Derived Products: A Review. Oxidative Med. Cell. Longev..

[154] Bordoloi S., Pathak K., Devi M., Saikia R., Das J., Kashyap V.H., Das D., Ahmad M.Z., Abdel-Wahab B.A. (2024). Some Promising Medicinal Plants Used in Alzheimer’s Disease: An Ethnopharmacological Perspective. Discov. Appl. Sci..

[155] Rudrapal M., Khairnar S.J., Khan J., Dukhyil A.B., Ansari M.A., Alomary M.N., Alshabrmi F.M., Palai S., Deb P.K., Devi R. (2022). Dietary Polyphenols and Their Role in Oxidative Stress-Induced Human Diseases: Insights Into Protective Effects, Antioxidant Potentials and Mechanism(s) of Action. Front. Pharmacol..

[156] Kumar G. (2022). A Review of the Chemical Constituents and Pharmacological Activities of Antirrhinum Majus (Snapdragon). IJCAAP.

[157] Saqallah F.G., Hamed W.M., Talib W.H. (2018). In Vivo Evaluation of Antirrhinum Majus’ Wound-Healing Activity. Sci. Pharm..

[158] Marques T.H.C., Melo C.H.S.D., Freitas R.M.D. (2012). In Vitro Evaluation of Antioxidant, Anxiolytic and Antidepressant-like Effects of the Bellis Perennis Extract. Rev. Bras. Farm..

[159] Bouallag O., Zeghichi-Hamri S., Bachir-Bey M., Brahmi-Chendouh N., Bouallag N., Takka M., Madani K., Boulekabache L. (2022). Optimization of Phenolic Compounds Extraction from Bellis Perennis Flowers and Assessment for Antioxidant Properties. AUDJG-Food Technol..

[160] Gollen B., Mehla J., Gupta P. (2018). Clitoria-Ternatea-Linn-a-Herb-with-Potential-Pharmacological-Activitiesfuture-Prospects-as-Therapeutic-Herbal-Medicine. J. Pharmacol. Rep..

[161] Phuse S.S., Khan Z.H. (2018). Evaluation for Antioxidant Potential and Hemolytic Effect of Cosmos Sulphureus Flower Extracts. J. Emerg. Technol. Innov. Res..

[162] Sharma Y. (2011). Health and Nutrition from Ornamentals. Int. J. Res. Ayurveda Pharm..

[163] Jang I.-C., Park J.-H., Park E., Park H.-R., Lee S.-C. (2008). Antioxidative and Antigenotoxic Activity of Extracts from Cosmos (Cosmos Bipinnatus) Flowers. Plant Foods Hum. Nutr..

[164] Sunjono T.A., Rahman A. (2020). Anti-Hypercholesterolemic Activity of Ethnolic Extract of Gomphrena Globosa Flowers and Its Phytochemical Screening. IJPR.

[165] Cichewicz R.H., Nair M.G. (2002). Isolation and Characterization of Stelladerol, a New Antioxidant Naphthalene Glycoside, and Other Antioxidant Glycosides from Edible Daylily (*Hemerocallis*) Flowers. J. Agric. Food Chem..

[166] Liu L., Yuan Y., Zuo J., Tao J. (2023). Composition and Antioxidant Activity of Paeonia Lactiflora Petal Flavonoid Extract and Underlying Mechanisms of the Protective Effect on H_2_O_2_-Induced Oxidative Damage in BRL3A Cells. Hortic. Plant J..

[167] Liu L., Yuan Y., Tao J. (2021). Flavonoid-Rich Extract of Paeonia Lactiflora Petals Alleviate d-Galactose-Induced Oxidative Stress and Restore Gut Microbiota in ICR Mice. Antioxidants.

[168] Uzelac M., Sladonja B., Šola I., Dudaš S., Bilić J., Famuyide I.M., McGaw L.J., Eloff J.N., Mikulic-Petkovsek M., Poljuha D. (2023). Invasive Alien Species as a Potential Source of Phytopharmaceuticals: Phenolic Composition and Antimicrobial and Cytotoxic Activity of Robinia Pseudoacacia L. Leaf and Flower Extracts. Plants.

[169] Sabudak T., Guler N. (2009). *Trifolium* L.—A Review on Its Phytochemical and Pharmacological Profile. Phytother. Res..

[170] Kolodziejczyk-Czepas J. (2012). Trifolium Species-Derived Substances and Extracts—Biological Activity and Prospects for Medicinal Applications. J. Ethnopharmacol..

[171] Gościniak A., Szulc P., Zielewicz W., Walkowiak J., Cielecka-Piontek J. (2023). Multidirectional Effects of Red Clover (Trifolium Pratense L.) in Support of Menopause Therapy. Molecules.

[172] Yang X.N., Kang S.C. (2012). In Vitro Antioxidant Activity of the Water and Ethanol Extracts of *Forsythia Koreana* Flowers. Nat. Product. Res..

[173] Kamkaen N., Wilkinson J.M. (2009). The Antioxidant Activity of *Clitoria Ternatea* Flower Petal Extracts and Eye Gel. Phytother. Res..

[174] Suganya G., Kumar P., Dheeba B., Sivakumar R. (2014). In Vitro Antidiabetic, Antioxidant and Anti-Inflammatory Activity of *Clitoria ternatea* L.. Int. J. Pharm. Pharm. Sci..

[175] Mohd Salleh R., Zarini A. (2013). Total Phenolic Compounds and Scavenging Activity in Clitoria Ternatea and Vitex Negundo Linn. Int. Food Res. J..

[176] Tsai J.-C., Chiu C.-S., Chen Y.-C., Lee M., Hao X.-Y., Hsieh M.-T., Kao C.-P., Peng W.-H. (2017). Hepatoprotective Effect of Coreopsis Tinctoria Flowers against Carbon Tetrachloride-Induced Liver Damage in Mice. BMC Complement. Altern. Med..

[177] Hamiduzzaman M., Azam A.Z. (2012). Antimicrobial, Antioxidant and Cytotoxic Activities of Gomphrena Globosa (L.). Bangla Pharma J..

[178] Cambay Z., Baydas G., Tuzcu M., Bal R. (2011). Pomegranate (*Punica granatum* L.) Flower Improves Learning and Memory Performances Impaired by Diabetes Mellitus in Rats. Acta Physiol. Hung..

[179] Mu H., Bai Y.-H., Wang S.-T., Zhu Z.-M., Zhang Y.-W. (2009). Research on Antioxidant Effects and Estrogenic Effect of Formononetin from Trifolium Pratense (Red Clover). Phytomedicine.

[180] Hou Y., Dan X., Babbar M., Wei Y., Hasselbalch S.G., Croteau D.L., Bohr V.A. (2019). Ageing as a Risk Factor for Neurodegenerative Disease. Nat. Rev. Neurol..

[181] Adamu A., Li S., Gao F., Xue G. (2024). The Role of Neuroinflammation in Neurodegenerative Diseases: Current Understanding and Future Therapeutic Targets. Front. Aging Neurosci..

[182] Moise G., Jîjie A.-R., Moacă E.-A., Predescu I.-A., Dehelean C.A., Hegheș A., Vlad D.C., Popescu R., Vlad C.S. (2024). Plants’ Impact on the Human Brain—Exploring the Neuroprotective and Neurotoxic Potential of Plants. Pharmaceuticals.

[183] Kataria P. (2024). Gourmet Gardens: The Edible Flower Revolution. Food Sci. Nutr. Cases.

[184] Karakaş P. (2011). Effects of Common Daisy (Bellis Perennis L.) Aqueous Extracts on Anxiety-like Behaviour and Spatial Memory Performance in Wistar Albino Rats. Afr. J. Pharm. Pharmacol..

[185] Khan A., Vaibhav K., Javed H., Khan M., Tabassum R., Ahmed M., Raza S., Ashafaq M., Khuwaja V. (2011). Neuroprotective Effect of Bellis Perennis and Hypericum Perforatum on PC12 Cells. Indian J. Res. Homoeopath..

[186] Marques T., Cardoso K., Almeida A., Tome A., Freitas R. (2011). Behavioral Studies and Histopathological Changes in Mice Pretreated with Bellis Perennis in Pilocarpine-Induced Seizures. Bol. Latinoam. Caribe Plantas Med. Aromat..

[187] Ma P., Zhang R., Xu L., Liu H., Xiao P. (2022). The Neuroprotective Effects of Coreopsis Tinctoria and Its Mechanism: Interpretation of Network Pharmacological and Experimental Data. Front. Pharmacol..

[188] Le L., Fu H., Lv Q., Bai X., Zhao Y., Xiang J., Jiang B., Hu K., Chen S. (2019). The Protective Effects of the Native Flavanone Flavanomarein on Neuronal Cells Damaged by 6-OHDA. Phytomedicine.

[189] Li N., Meng D., Pan Y., Cui Q., Li G., Ni H., Sun Y., Qing D., Jia X., Pan Y. (2015). Anti-Neuroinflammatory and NQO1 Inducing Activity of Natural Phytochemicals from Coreopsis Tinctoria. J. Funct. Foods.

[190] Wu Z., Chen L., Guo Z., Li K., Fu Y., Zhu J., Chen X., Huang C., Zheng C., Ma Y. (2019). Systems Pharmacology Uncovers Serotonergic Pathway Mediated Psychotherapeutic Effects of Lonicerae Japonicae Flos. J. Funct. Foods.

[191] Echeverria V., Echeverria F., Barreto G.E., Echeverría J., Mendoza C. (2021). Estrogenic Plants: To Prevent Neurodegeneration and Memory Loss and Other Symptoms in Women After Menopause. Front. Pharmacol..

[192] Fu X., Qin T., Yu J., Jiao J., Ma Z., Fu Q., Deng X., Ma S. (2019). Formononetin Ameliorates Cognitive Disorder via PGC-1α Pathway in Neuroinflammation Conditions in High-Fat Diet-Induced Mice. CNSNDDT.

[193] Howes J.B., Bray K., Lorenz L., Smerdely P., Howes L.G. (2004). The Effects of Dietary Supplementation with Isoflavones from Red Clover on Cognitive Function in Postmenopausal Women. Climacteric.

[194] Chopra D., Shukla S., Rana P., Kamar M.D., Gaur P., Bala M., Pathaniya D., Tripathi A., Dwivedi A., Gupta S., Poojan S. (2024). Overview of Inflammation. Inflammation Resolution and Chronic Diseases.

[195] Rainsford K.D., Harris R.E., Bittman R., Dasgupta D., Engelhardt H., Flohe L., Herrmann H., Holzenburg A., Nasheuer H.-P., Rottem S., Wyss M. (2007). Anti-Inflammatory Drugs in the 21st Century. Inflammation in the Pathogenesis of Chronic Diseases.

[196] Chen Q., Xu B., Huang W., Amrouche A.T., Maurizio B., Simal-Gandara J., Tundis R., Xiao J., Zou L., Lu B. (2020). Edible Flowers as Functional Raw Materials: A Review on Anti-Aging Properties. Trends Food Sci. Technol..

[197] Tasneem S., Liu B., Li B., Choudhary M.I., Wang W. (2019). Molecular Pharmacology of Inflammation: Medicinal Plants as Anti-Inflammatory Agents. Pharmacol. Res..

[198] Li Y., Cai W., Weng X., Li Q., Wang Y., Chen Y., Zhang W., Yang Q., Guo Y., Zhu X. (2015). Lonicerae Japonicae Flos and Lonicerae Flos: A Systematic Pharmacology Review. Evid.-Based Complement. Altern. Med..

[199] Liu C., Yin Z., Feng T., Zhang M., Zhou Z., Zhou Y. (2021). An Integrated Network Pharmacology and RNA-Seq Approach for Exploring the Preventive Effect of Lonicerae Japonicae Flos on LPS-Induced Acute Lung Injury. J. Ethnopharmacol..

[200] Parker S., May B., Zhang C., Zhang A.L., Lu C., Xue C.C. (2016). A Pharmacological Review of Bioactive Constituents of *Paeonia lactiflora* Pallas and *Paeonia veitchii* Lynch: Review of *P. lactiflora* Pallas and *P. veitchii* Lynch. Phytother. Res..

[201] Chen Y., Li H., Zhang X.-L., Wang W., Rashed M.M.A., Duan H., Li L.-L., Zhai K.-F. (2024). Exploring the Anti-Skin Inflammation Substances and Mechanism of Paeonia Lactiflora Pall. Flower via Network Pharmacology-HPLC Integration. Phytomedicine.

[202] Rashid M., Shafi S. (2018). Evaluation of Phytochemical Constituents and In Vitro Anti-Inflammatory Activity of Kashmiri Pomegranate (Punica Granatum Linn.) Flower Extract. IOSR J. Pharm. Biol. Sci..

[203] Xu J., Zhao Y., Aisa H.A. (2017). Anti-Inflammatory Effect of Pomegranate Flower in Lipopolysaccharide (LPS)-Stimulated RAW264.7 Macrophages. Pharm. Biol..

[204] Gheith I., El-Mahmoudy A. (2017). Potent Anti-Oxidant and Anti-Inflammatory Potentials of Punica Granatum Leaf and Flower Hydromethanolic Extracts in Vitro. Biosci. J..

[205] Lee S.G., Brownmiller C.R., Lee S.-O., Kang H.W. (2020). Anti-Inflammatory and Antioxidant Effects of Anthocyanins of Trifolium Pratense (Red Clover) in Lipopolysaccharide-Stimulated RAW-267.4 Macrophages. Nutrients.

[206] Hou Y., Li G., Wang J., Pan Y., Jiao K., Du J., Chen R., Wang B., Li N. (2017). Okanin, Effective Constituent of the Flower Tea Coreopsis Tinctoria, Attenuates LPS-Induced Microglial Activation through Inhibition of the TLR4/NF-κB Signaling Pathways. Sci. Rep..

[207] Saqallah F.G., Hamed W.M., Talib W.H., Dianita R., Wahab H.A. (2022). Antimicrobial Activity and Molecular Docking Screening of Bioactive Components of Antirrhinum Majus (Snapdragon) Aerial Parts. Heliyon.

[208] Tatli Cankaya I.I., Somuncuoglu E.I. (2021). Potential and Prophylactic Use of Plants Containing Saponin-Type Compounds as Antibiofilm Agents against Respiratory Tract Infections. Evid.-Based Complement. Altern. Med..

[209] Kusmiati K., Priadi D., Rahayu R.K.R. (2017). Antibacterial Activity Test, Evaluation of Pharmacognosy and Phytochemical Screening of Some Extracts of Globe Amaranth (Gomphrena Globosa). J. Pure Appl. Chem. Res..

[210] Ma S.-C., Du J., But P.P.-H., Deng X.-L., Zhang Y.-W., Ooi V.E.-C., Xu H.-X., Lee S.H.-S., Lee S.F. (2002). Antiviral Chinese Medicinal Herbs against Respiratory Syncytial Virus. J. Ethnopharmacol..

[211] Karakas F.P., Yidirim A.B., Bayram R., Yavuz M.Z., Gepdiremen A., Turker A.U. (2015). Antiproliferative Activity of Some Medicinal Plants on Human Breast and Hepatocellular Carcinoma Cell Lines and Their Phenolic Contents. Trop. J. Pharm. Res..

[212] Ninomiya K., Motai C., Nishida E., Kitagawa N., Yoshihara K., Hayakawa T., Muraoka O., Li X., Nakamura S., Yoshikawa M. (2016). Acylated Oleanane-Type Triterpene Saponins from the Flowers of Bellis Perennis Show Anti-Proliferative Activities against Human Digestive Tract Carcinoma Cell Lines. J. Nat. Med..

[213] Wufuer Y., Yang X., Guo L., Aximujiang K., Zhong L., Yunusi K., Wu G. (2022). The Antitumor Effect and Mechanism of Total Flavonoids From Coreopsis Tinctoria Nutt (Snow Chrysanthemum) on Lung Cancer Using Network Pharmacology and Molecular Docking. Front. Pharmacol..

[214] Chen X., Zhou X., Gao Y. (2024). Optimizing *Coreopsis tinctoria* Flower Extraction and Inhibiting CML Activity: Box-Behnken Design. ACAMC.

[215] Luo F., Manse Y., Ishikawa S., Nishi S., Chen A., Wang T., Morikawa T. (2023). Aromatase Inhibitors Isolated from a Flowering Tea, Snow Chrysanthemum (the Capitula of Coreopsis Tinctoria Nutt.). J. Nat. Med..

[216] Bratu M.M., Birghila S., Stancu L.M., Cenariu M.C., Emoke P., Popescu A., Radu M.D., Zglimbea L. (2021). Evaluation of the Antioxidant, Cytotoxic and Antitumoral Activities of a Polyphenolic Extract of Robinia Pseudoacacia L. Flowers. J. Sci. Arts.

[217] Manzoureh R., Farahpour M.R. (2021). Topical Administration of Hydroethanolic Extract of *Trifolium pratense* (Red Clover) Accelerates Wound Healing by Apoptosis and Re-Epithelialization. Biotech. Histochem..

[218] Zangeneh M.M., Zangeneh A., Tahvilian R., Moradi R., Tehrani P.R. (2018). Preclinical Evaluation of Hematoprotective and Nephroprotective Activities of Bellis Perennis L. Aqueous Extract on CCl4-Induced Renal Injury in Mice. Comp. Clin. Pathol..

[219] Deepika B.K., Apoorva N.H., Joel P.R., Sudheer S.P. (2024). Enhanced Osteogenic Differentiation Potential of Arnica Montana and Bellis Perennis in C3H10T1/2 Multipotent Mesenchymal Stem Cells. Mol. Biol. Rep..

[220] Struckmann V.F., Allouch-Fey S., Kneser U., Harhaus L., Schulte M. (2024). Indication-Specific Effect of a Phytotherapeutic Remedy on Human Fetal Osteoblastic Cells: An in Vitro Analysis. Complement. Med. Res..

[221] Haselgrübler R., Stadlbauer V., Stübl F., Schwarzinger B., Rudzionyte I., Himmelsbach M., Iken M., Weghuber J. (2018). Insulin Mimetic Properties of Extracts Prepared from Bellis Perennis. Molecules.

[222] Yao L., Li L., Li X., Li H., Zhang Y., Zhang R., Wang J., Mao X. (2015). The Anti-Inflammatory and Antifibrotic Effects of Coreopsis Tinctoria Nutt on High-Glucose-Fat Diet and Streptozotocin-Induced Diabetic Renal Damage in Rats. BMC Complement. Altern. Med..

[223] Yang Q., Sun Y., Zhang L., Xu L., Hu M., Liu X., Shi F., Gu Z. (2014). Antihypertensive Effects of Extract from Flower Buds of Coreopsis Tinctoria on Spontaneously Hypertensive Rats. Chin. Herbal. Med..

[224] Sun Y.-H., Zhao J., Jin H.-T., Cao Y., Ming T., Zhang L.-L., Hu M.-Y., Hamlati H., Pang S.-B., Ma X.-P. (2013). Vasorelaxant Effects of the Extracts and Some Flavonoids from the Buds of *Coreopsis tinctoria*. Pharm. Biol..

[225] Fathima S.N., Murthy S.V. (2019). Cardioprotective Effects to Chronic Administration of Gomphrena Globosa Flowers in Isoproterenol Induced Myocardial Infarction: Biochemical, Histopathological and Ultrastructural Studies. IJPR.

[226] Huang T., Peng G., Kota B., Li G., Yamahara J., Roufogalis B., Li Y. (2005). Anti-Diabetic Action of Flower Extract: Activation of PPAR-γ and Identification of an Active Component. Toxicol. Appl. Pharmacol..

[227] Nasiri E., Hosseinimehr S.J., Akbari J., Azadbakht M., Azizi S. (2017). The Effects of *Punica granatum* Flower Extract on Skin Injuries Induced by Burn in Rats. Adv. Pharmacol. Sci..

[228] Mahmoudieh M., Keleidari B., Nasr Esfahani F., Zolfaghari B., Melali H., Davarpanah Jazi A.H., Mehdinezhad N., Mokhtari M. (2020). The Effect of Punica Granatum L. Flower Extract on Post-Surgical Peritoneal Adhesions in a Rat Model. Eur. J. Obstet. Gynecol. Reprod. Biol..

[229] Saeidnia S., Manayi A., Abdollahi M. (2016). From in Vitro Experiments to in Vivo and Clinical Studies; Pros and Cons. CDDT.

[230] Mitchell M.J., Billingsley M.M., Haley R.M., Wechsler M.E., Peppas N.A., Langer R. (2021). Engineering Precision Nanoparticles for Drug Delivery. Nat. Rev. Drug Discov..

[231] Madorran E., Stožer A., Bevc S., Maver U. (2019). In Vitro Toxicity Model: Upgrades to Bridge the Gap between Preclinical and Clinical Research. Bosn. J. Basic. Med. Sci..

[232] Pound P., Ritskes-Hoitinga M. (2018). Is It Possible to Overcome Issues of External Validity in Preclinical Animal Research? Why Most Animal Models Are Bound to Fail. J. Transl. Med..

